# Myocardial ATP depletion detected noninvasively predicts sudden cardiac death risk in patients with heart failure

**DOI:** 10.1172/jci.insight.157557

**Published:** 2022-06-22

**Authors:** T. Jake Samuel, Shenghan Lai, Michael Schär, Katherine C. Wu, Angela M. Steinberg, An-Chi Wei, Mark E. Anderson, Gordon F. Tomaselli, Gary Gerstenblith, Paul A. Bottomley, Robert G. Weiss

**Affiliations:** 1 Division of Cardiology, Department of Medicine, Johns Hopkins University School of Medicine, Baltimore, Maryland, USA.; 2 Institute of Human Virology, University of Maryland School of Medicine, Baltimore, Maryland, USA.; 3 Department of Epidemiology, Johns Hopkins Bloomberg School of Public Health, Baltimore, Maryland, USA.; 4 Division of Magnetic Resonance Research, Department of Radiology and Radiological Science, Johns Hopkins University School of Medicine, Baltimore, Maryland, USA.; 5 Department of Electrical Engineering, Graduate Institute of Biomedical Electronics and Bioinformatics, National Taiwan University, Taipei, Taiwan.; 6 Department of Physiology, Program in Cellular and Molecular Medicine, and Department of Genetic Medicine, Johns Hopkins University School of Medicine, Baltimore, Maryland, USA.; 7 Division of Cardiology, Department of Medicine, Albert Einstein College of Medicine, Bronx, New York, USA.

**Keywords:** Cardiology, Arrhythmias, Bioenergetics, Heart failure

## Abstract

**BACKGROUND:**

Sudden cardiac death (SCD) remains a worldwide public health problem in need of better noninvasive predictive tools. Current guidelines for primary preventive SCD therapies, such as implantable cardioverter defibrillators (ICDs), are based on left ventricular ejection fraction (LVEF), but these guidelines are imprecise: fewer than 5% of ICDs deliver lifesaving therapy per year. Impaired cardiac metabolism and ATP depletion cause arrhythmias in experimental models, but to our knowledge a link between arrhythmias and cardiac energetic abnormalities in people has not been explored, nor has the potential for metabolically predicting clinical SCD risk.

**METHODS:**

We prospectively measured myocardial energy metabolism noninvasively with phosphorus magnetic resonance spectroscopy in patients with no history of significant arrhythmias prior to scheduled ICD implantation for primary prevention in the setting of reduced LVEF (≤35%).

**RESULTS:**

By 2 different analyses, low myocardial ATP significantly predicted the composite of subsequent appropriate ICD firings for life-threatening arrhythmias and cardiac death over approximately 10 years. Life-threatening arrhythmia risk was approximately 3-fold higher in patients with low ATP and independent of established risk factors, including LVEF. In patients with normal ATP, rates of appropriate ICD firings were several-fold lower than reported rates of ICD complications and inappropriate firings.

**CONCLUSION:**

To the best of our knowledge, these are the first data linking in vivo myocardial ATP depletion and subsequent significant arrhythmic events in people, suggesting an energetic component to clinical life-threatening ventricular arrhythmogenesis. The findings support investigation of metabolic strategies that limit ATP loss to treat or prevent life-threatening cardiac arrhythmias and herald noninvasive metabolic imaging as a complementary SCD risk stratification tool.

**TRIAL REGISTRATION:**

ClinicalTrials.gov NCT00181233.

**FUNDING:**

This work was supported by the DW Reynolds Foundation, the NIH (grants HL61912, HL056882, HL103812, HL132181, HL140034), and Russell H. Morgan and Clarence Doodeman endowments at Johns Hopkins.

## Graphical abstract

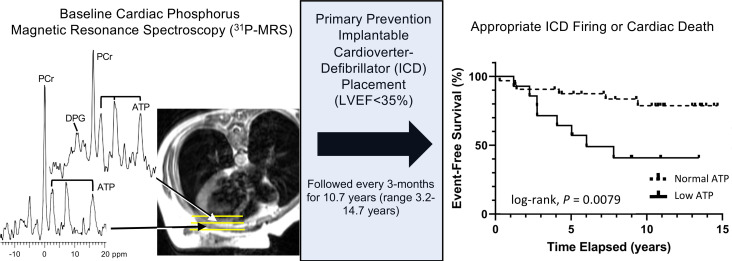

## Introduction

Sudden cardiac death (SCD) is a major worldwide health care problem accounting for approximately 50% of all cardiovascular deaths and claiming more than 300,000 American lives annually (1, 2). Although primary prevention implantable cardioverter defibrillators (ICDs) reduce SCD in patients with reduced left ventricular ejection fraction (LVEF), appropriate annual ICD firing rates are low (~5% per year), and most devices do not deliver lifesaving therapy during the device lifetime. In addition, ICDs are costly and associated with procedural and postprocedural risks (3, 4). Thus, noninvasive approaches that better assess SCD risk are needed (1, 5).

Although genetic, structural, electrophysiological, and clinical factors all contribute to the underlying substrate and triggers that result in SCD (6), impaired myocardial metabolism may represent a common but inadequately studied mechanism linking these risk factors to accentuated SCD risk, especially in people with heart failure or prior myocardial infarction. Impaired myocardial ATP metabolism is a consistent finding in preclinical heart failure models and in human heart failure due to common etiologies (7, 8). Moreover, in patients with heart failure, left ventricular structural factors associated with increased SCD risk (e.g., ventricular dysfunction and pathologic remodeling) are associated with cardiac energetic abnormalities (7, 9, 10). In experimental models, metabolic inhibition and/or ischemia-mediated metabolic stress per se alter myocyte excitability and arrhythmias via altered activity of energy-dependent cardiac ion channels (11, 12). Moreover, low intracellular Mg-ATP directly increases arrhythmic susceptibility, and ATP administration counteracts this effect, highlighting the proarrhythmic effect of low ATP concentrations (13). Despite a significant body of literature supporting a role for impaired mitochondrial metabolism and ATP depletion in experimental arrhythmogenesis (14), to date there are no studies of cardiac ATP metabolism and SCD risk in people.

Human myocardial high-energy phosphate levels and rates of ATP synthesis through creatine kinase (CK), the primary energy reserve reaction of the heart, can be measured noninvasively using phosphorus magnetic resonance spectroscopy (^31^P MRS) with clinical MR scanners. As part of the Prospective Observational Study of the ICD in Sudden Cardiac Death Prevention (PROSe-ICD) (15), we prospectively performed cardiac ^31^P MRS prior to clinically indicated, primary prevention ICD implantation in patients with an LVEF of 35% or less (ClinicalTrials.gov NCT00181233). Participants were subsequently followed with semiannual visits and ICD interrogations for approximately 10 years, and an independent committee of board-certified electrophysiologists blinded to all clinical, biomarker, and cardiac magnetic resonance (CMR) data adjudicated all ICD firings and deaths.

To test the central hypothesis that impaired myocardial high-energy phosphate metabolism predicts SCD risk as assessed by appropriate ICD firings and/or cardiac death, we adopted 2 analysis approaches that ultimately gave similar results. First, we used a conventional time to first event approach, and second, we used multiple event analysis (MEA). MEA offers additional rigor and is clinically relevant because multiple ICD firings are associated with increased hospitalization and mortality rates (16).

## Results

**Figure 1:**
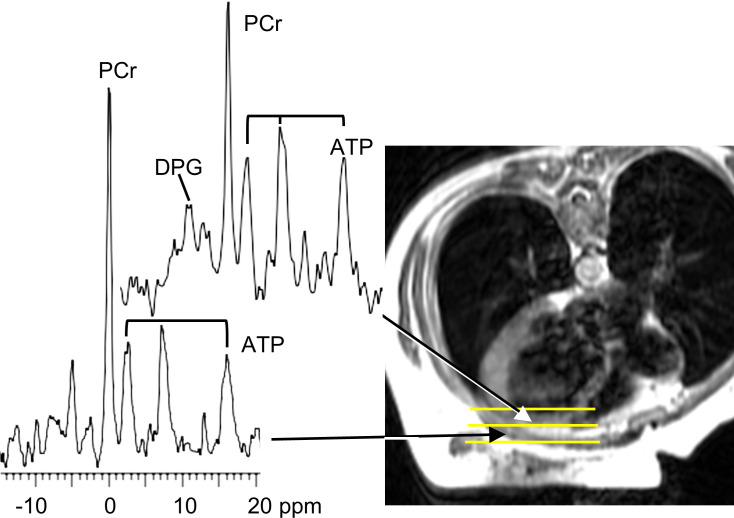
Representative CMR image and ^31^P MR spectra. Representative axial MRI (right) of the chest of a 45-year-old woman (lying prone) who subsequently experienced an arrhythmic event. Yellow lines denote localized volumes from which ^31^P MRS spectra (left) were derived (arrows). The upper spectrum from the heart shows phosphocreatine (PCr), diphosphoglycerate (DPG), and the 3 phosphate resonances of ATP. The myocardial ATP was low (2.5 μmol/g wet wt) but PCr/ATP was normal (1.7) in this individual. The lower spectrum includes chest muscle and is shown for comparison but was not used in analysis.

We studied 46 participants (52 ± 13 years, mean ± SD), who were predominantly male (57%) and White (74%). Two-thirds of participants had nonischemic cardiomyopathy (63%), and 17% had type 2 diabetes (Table 1 and Supplemental Tables 1 and 2; supplemental material available online with this article; https://doi.org/10.1172/jci.insight.157557DS1). The mean LVEF by MRI was 28% ± 9%; end-diastolic volume, 223 ± 54 mL; cardiac phosphocreatine (PCr)/ATP, 1.79 ± 0.51; [PCr], 7.44 ± 2.63 μmol/g; [ATP], 4.31 ± 1.43 μmol/g; and CK flux, 1.81 ± 1.00 μmol/g/s. Representative ^31^P MR spectra are shown in Figure 1.

**Figure 2:**
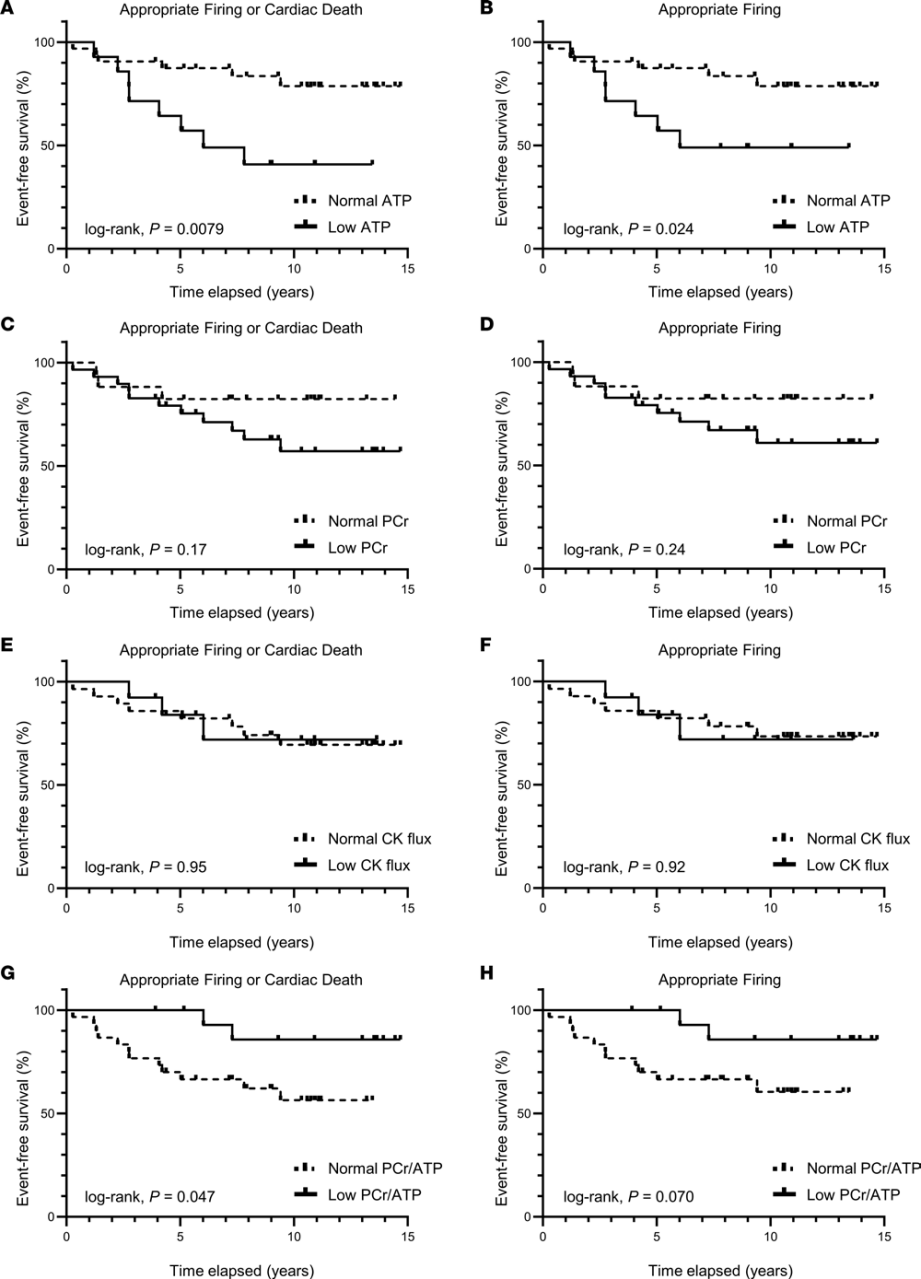
Event-free survival in patients with low versus normal myocardial energetics. Kaplan-Meier curves depicting the proportion of event-free survival across a median follow-up period of 10.7 years (range: 3.2–14.7 years) in individuals with low (solid line) versus individuals with normal (dashed line) myocardial high-energy phosphates. We defined low myocardial energetics as 2 standard deviations below the grouped mean of healthy individuals previously reported by our group (ATP: <3.4 μmol/g; PCr: <7.6 μmol/g; CK flux: <1.3 μmol/g/s) (7, 9). Cardiac PCr/ATP cut point was less than 1.6, as previously reported (8). (**A**, **C**, **E**, and **G**) Event-free survival is shown where the event was defined as the composite endpoint of appropriate ICD firing or cardiac death. (**B**, **D**, **F**, and **H**) Event-free survival is shown where the event was defined as appropriate ICD firing only. Low myocardial ATP concentration was associated with lower event-free survival when considering the composite endpoint (log-rank, *P* = 0.0079) or appropriate ICD firing alone (log-rank, *P* = 0.024). Neither low PCr or low CK flux were significant predictors of event-free survival (log rank, *P* > 0.05). (**A** and **B**) *n* = 32 versus 14 (normal versus low ATP); (**C** and **D**) *n* = 17 versus 29 (normal versus low PCr); (**E** and **F**) *n* = 28 versus 13 (normal versus low CK flux); (**G** and **H**) *n* = 30 versus 16 (normal versus low PCr/ATP).

During a median follow-up period of 10.7 years (range 3.2–14.7 years), 30.4% of the cohort experienced at least 1 appropriate ICD firing and 13.0% had cardiac death. For the composite endpoint of appropriate ICD firing or cardiac death, conventional time to first event analysis showed that low myocardial ATP concentration (<3.4 μmol/g), as defined by values 2 standard deviations below the grouped mean of contemporaneous healthy controls (7, 9), was associated with a significantly lower probability of event-free survival (log-rank, *P* = 0.0079; Figure 2A). Indeed, only 41% of the participants with low ATP concentration at baseline were event free at the 10-year time point, in comparison with 79% of the participants with normal ATP concentrations. In contrast, reduced cardiac PCr concentration, PCr/ATP, or CK flux were not associated with an increased risk of arrhythmic event (appropriate ICD firing or cardiac death, Figure 2, C, E, and G).

For the second approach, we performed MEA for the composite of appropriate ICD firings and/or cardiac death. Five individuals (10.9%) had 1 event, 5 individuals (10.9%) had 2 events, none (0.0%) had 3 events, 1 individual (2.2%) had 4 events, and 3 individuals (6.5%) had 5 events. MEA showed that individuals with low cardiac ATP concentrations at baseline were at a 2.98-fold greater risk of having multiple appropriate ICD firings or cardiac death compared with individuals with normal ATP concentrations (Table 2). Note that the ability of low ATP to predict the composite endpoint by MEA was independent of well-established SCD risk factors, including LVEF, sex, age, and race (HR: 2.98, 95% CI: 1.27–6.99, *P* = 0.01; Table 2). Similar to the time to first event analysis, low PCr and CK flux were not independent predictors for multiple events by MEA (both *P* > 0.05).

Although the composite endpoint of appropriate ICD firings or cardiac death is clinically relevant, cardiac deaths may include nonarrhythmic deaths. Therefore, we repeated the analysis using only appropriate ICD firings. Again, conventional time to first event analysis showed that low myocardial ATP concentration was associated with a significantly higher probability of appropriate ICD firings (log-rank, *P* = 0.024; Figure 2B), whereas cardiac PCr concentration, low PCr/ATP, and CK flux were not (*P* > 0.05; Figure 2, D, F, and H). MEA for appropriate ICD firings also showed that individuals with low cardiac ATP concentrations at baseline were at a 3.36-fold greater risk of having multiple appropriate ICD firings compared with individuals with normal ATP concentrations (Table 3).

To gain further insight into the potential causes of low cardiac ATP, we compared demographics, comorbidities, and biomarker data in the subgroup with low cardiac ATP versus the subgroup with normal ATP (Supplemental Table 2). There were no significant differences in age, race, sex, medications, comorbidities (hypertension, hypercholesterolemia, tobacco abuse), left bundle branch block, renal function, electrolytes, or serum inflammatory biomarkers between the low and normal ATP groups, although there were more individuals with diabetes in the low ATP group (*P* = 0.03, Supplemental Table 2). In terms of heart failure metrics, there were also no significant differences in New York Heart Association (NYHA) class, cardiomyopathy duration, etiology, LVEF, and left ventricular volumes between the low and normal ATP cohorts. However, N-terminal pro–brain natriuretic peptide (NT-proBNP) was higher in the low ATP group (*P* = 0.043). Thus, many of the demographic and clinical metrics that were previously associated with worse arrhythmic, cardiovascular, or noncardiovascular outcomes in patients with reduced ejection fraction were not different in the cohort with low cardiac ATP as compared with individuals with normal cardiac ATP.

To test whether electrical or electrophysiological indices differed between the low and normal ATP subgroups, we compared ECG metrics and the results of electrophysiological testing at baseline. There were no significant differences in inducible ventricular arrhythmias, QRS duration, QT duration, heart rate variability, or the signal-averaged ECG between the low and normal ATP groups (Supplemental Table 2).

**Figure 3:**
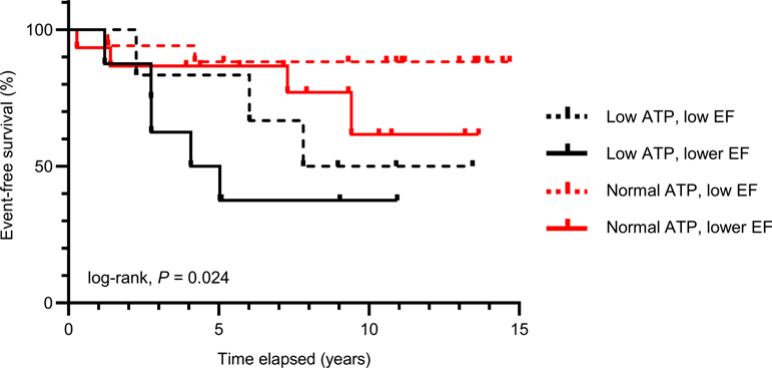
Event-free survival based on cardiac ATP and LVEF. Kaplan-Meier curves depicting the proportion of event-free survival across a median follow-up period of 10.7 years (range: 3.2–14.7 years) in individuals grouped by myocardial ATP (normal versus low ATP, <3.4 μmol/g) and by LVEF (low LVEF versus lower LVEF, <28% based on group median). The 2 subgroups with normal ATP (red lines) had the lowest arrhythmic risk, and the 2 subgroups with low ATP (black lines) had the highest risk, regardless of whether they had low ejection fraction (dotted lines) or lower ejection fraction (solid lines; log-rank, *P* = 0.024). The lowest arrhythmic risk subgroup had normal ATP and less depressed LVEF and exhibited nearly a 90% event-free survival at 10–15 years. The highest arrhythmic risk subgroup had both low ATP and the lowest LVEF, with a 5-year event-free survival of only approximately 35%. Low ATP, low ejection fraction *n* = 6; low ATP, lower ejection fraction *n* = 8; normal ATP, low ejection fraction *n* = 17; normal ATP, lower ejection fraction *n* = 15. The log-rank *P* value reflects the overall comparison of the 4 groups, and a significant *P* value rejects the null hypothesis that the groups are identical.

Given the central role of low LVEF in ventricular arrhythmia prediction in current clinical guidelines for primary prevention ICD implantation, we next investigated relationships between cardiac ATP and LVEF. In this entire study population, there was no significant relationship between ATP and LVEF (Supplemental Figure 1) and no difference in ejection fraction between the low and normal ATP groups (Supplemental Table 2). Further, when arrhythmic risk was investigated based on both ATP and ejection fraction (Figure 3), the subgroups with normal ATP had the lowest arrhythmic risk (both low ejection fraction and lower ejection fraction subgroups) and the 2 subgroups with low ATP had highest risk (both low ejection fraction and lower ejection fraction subgroups). The lowest arrhythmic risk subgroup had normal ATP and better LVEF and exhibited nearly a 90% event-free survival at 10–15 years. The highest arrhythmic risk subgroup was the group with both low ATP and the lowest LVEF, whose 5-year event-free survival was only approximately 35%.

Finally, we determined whether the myocardial free energy of ATP hydrolysis (Gibbs free energy of ATP hydrolysis, ΔG_~ATP_), which fuels the energy-dependent pumps that maintain myocyte ionic balance, was altered. We observed that myocardial ΔG_~ATP_ was less favorable at baseline in patients with low ATP compared with patients with normal ATP (59.5 ± 0.8 kJ/mol vs. 61.4 ± 2.3 kJ/mol, *P* = 0.0002; low vs. normal ATP, respectively; Supplemental Table 2).

## Discussion

These data demonstrate a link between impaired in vivo myocardial energetics, namely ATP depletion, and subsequent life-threatening ventricular arrhythmic events in people. In MEA, the ventricular arrhythmic risk associated with low cardiac ATP was approximately 3-fold higher than that in individuals with normal ATP and independent of established risk factors, including low LVEF. These findings in a high-risk patient population are consistent with prior preclinical data demonstrating a mechanistic role for impaired cardiac metabolism in experimental arrhythmogenesis. The current findings are an important first step in recognizing the metabolic-energetic aspects of life-threatening arrhythmogenesis in people.

Prior preclinical work demonstrated that metabolic inhibition and ATP depletion can cause both triggered and reentrant arrhythmias. Maintenance of intracellular ionic concentrations is critical for preventing arrhythmias, and the Na^+^/K^+^ ATPase and the sarco/endoplasmic reticulum Ca^++^-ATPase (SERCA) are 2 of the most important. Both are driven by ΔG_~ATP_ (14). Disruption of these energy-dependent gradients increases the risk of ventricular fibrillation, triggers extrasystoles, and alters refractoriness (12, 14, 17–20). In addition, local mitochondrial and high-energy phosphate abnormalities have been linked to spatiotemporal heterogeneity in the cardiac action potential that also predispose to reentrant arrhythmias (21). Finally, local mitochondrial dysfunction increases production of ROS that result in “metabolic sinks” that cause regions of conduction block and create yet another substrate for reentrant arrhythmias (22, 23). Thus, ATP depletion and metabolic abnormalities are mechanistic substrates for both triggered and reentrant experimental arrhythmias and may also contribute to arrhythmogenesis during times of acute metabolic stress.

Because the patients with low myocardial ATP levels did not exhibit differences in electrocardiographic or inducible ventricular arrhythmias at baseline as compared with patients with normal ATP (Supplemental Table 2) but later were more likely to develop life-threatening arrhythmias (Figures 2 and 3; Tables 2 and 3), we hypothesize that low cardiac ATP is a “substrate” for arrhythmogenesis (6) and that subsequent stressors or increased metabolic demand, due to physical or emotional stress or to induced-ischemia, may further jeopardize energetic balance and “trigger” arrhythmias in patients with already low ATP levels.

To gain some initial insight into the potential electrophysiological impact of energetic abnormalities like those observed in these patients, we explored a well-established computational model that incorporates mitochondrial bioenergetics and electrophysiology in the ventricular myocyte (24). When the energetic parameters measured in these patients were inserted in the computational model with all other input parameters held constant between the normal and low ATP groups, we observed that the action potential duration was modestly prolonged and that SERCA flux was reduced in low ATP conditions compared with normal ATP conditions (Supplemental Figure 3). A theoretical increase in action potential duration or heterogeneity could contribute to reentrant ventricular arrhythmias, and reduced SERCA could alter calcium handling and refractoriness, in turn increasing the propensity for triggered arrhythmias. It is worth noting that SERCA has one of the highest energy requirements (ΔG_~ATP_) of the ATPase reactions (25, 26) and that we observed a significantly reduced ΔG_~ATP_ in the patients with low ATP versus normal ATP (*P* = 0.0002; low vs. normal ATP, respectively; Supplemental Table 2). It is important to acknowledge that this computational analysis uses many input parameters that were not determined in these patients. Nevertheless, the ionic and current input parameters in the simulations are those used in the literature, treat the low and normal ATP groups the same, and now incorporate cardiac energetic parameters measured in these patients. As such, the simulations indicate that the degree of ATP depletion observed in these patients alone would prolong the action potential and impair calcium handling, 2 established ionic mechanisms contributing to ventricular reentry and triggered arrhythmias.

Future trials are needed to determine whether metabolic modulators or interventions that preserve or augment cardiac ATP reduce the risk of life-threatening arrhythmias in high-risk patients. Nevertheless, it is worth noting today that some conventional antiarrhythmic drugs (class I and III antiarrhythmics) neither alter energy metabolism nor reduce SCD risk, while medications that reduce energetic demand (beta blockers, aldosterone antagonists), also reduce SCD risk (27). Of note, there is emerging evidence that metabolic modulators like the new SGLT2 inhibitors reduce arrhythmias in animal models (28) and SCD risk in patients with reduced ejection fraction (29). Taken together, our observations of an independent relationship between myocardial ATP depletion and life-threatening arrhythmias in people build on prior basic scientific discovery and frame clinical ventricular arrhythmogenesis, in part, as a metabolic disease. They offer a noninvasive means to both identify increased metabolic arrhythmic risk and to determine the energetic consequences of metabolic interventions. Most importantly, these findings provide a foundation for future investigations of the antiarrhythmic potential of metabolic modulators.

Although a significant association of low myocardial ATP concentrations with SCD risk does not prove causality, detection of cardiac ATP depletion by ^31^P MRS offers a noninvasive metabolic means to identify increased SCD risk that may complement more established anatomic and functional SCD risk factors like LVEF, ventricular remodeling, and fibrosis/scarring measured by MRI on the same MRI scanners. Among biomarkers for SCD risk, reduced LVEF is one of the strongest and is a central clinical criterion used today for primary prevention ICD implantation. We show here that in patients with reduced LVEF who qualified for primary prevention ICD implantation, a low myocardial ATP level predicted individuals who were most likely to have an appropriate ICD firing or cardiac death and that independent association remained significant after adjusting for LVEF (Table 2 and Table 3). Further, in this entire study population of patients with low LVEF, ATP concentration did not correlate with ejection fraction (Supplemental Figure 1), mean LVEF was similar in the low ATP and normal ATP cohorts (Supplemental Table 2), and LVEF changed over the first 3 years after ICD implant in a similar fashion in the low and normal ATP groups (Supplemental Figure 2). Further, when both ATP and LVEF were dichotomized, ATP and LVEF were complementary, and low ATP still distinguished lower from higher SCD risks (Figure 3). From a risk prediction perspective, conventional demographic and clinical indices were not strong surrogates for distinguishing low from normal cardiac ATP levels (Supplemental Table 2), with the caveat that diabetes (*P* = 0.03), ischemic etiology (*P* = 0.061), and higher NT-proBNP (*P* = 0.043) tended to be associated with lower ATP, as might be expected.

More work is needed to replicate these initial findings in larger, more diverse populations and to better define the ultimate impact on clinical practice and risk prediction. Although it may be premature today to defer ICD placement in a patient with normal myocardial ATP levels based on this single report, it is encouraging to observe that only approximately 20% of the patients with normal cardiac ATP studied here had an appropriate ICD firing or cardiac death over 10–15 years (Figure 2), resulting in an annualized combined event rate of 1.3%–2.0% per year. Although ICD complications are more common in the first year after implantation, complications can and do occur years later. For the purpose of comparison, it is worth noting that the average rates of appropriate ICD firings in patients with normal cardiac ATP concentrations observed here (1.3%–2.0%) are much lower than the approximately 4% average annual rates of ICD complications (30) and the 2.5%–11% annual rates of inappropriate ICD firings (31). In addition, the annual arrhythmic rates are even lower (0.8%–1.0% per year) for individuals with normal ATP and higher LVEF (Figure 3). In the future, ATP levels may be one factor to consider when balancing the benefits and competing risks over 10+ years of ICD placement, especially considering the typical battery lifetime of ICDs. Arguably, the most relevant clinical time horizon for assessing SCD risk as part of the decision matrix to implant an initial ICD tends to be about 5–7 years, the lifetime of most ICDs (32). It is thus reassuring that the Kaplan-Meier curves for ATP prediction began to diverge within this window at about 2–2.5 years (Figure 2, A and B). It would have been difficult to detect differences sooner in this cohort, given the low number of early events in the first year.

Impaired myocardial CK metabolism, but not ATP depletion, was shown previously to predict heart failure events, including heart failure hospitalizations, left ventricular assist device implantation, and transplantation (33). Those observations are consistent with murine heart failure studies showing that restoration of reduced myofibrillar CK metabolism improves contractile indices, consistent with the energy reserve role of CK (34). Clinical heart failure outcomes differ from arrhythmic outcomes, and it is therefore perhaps not surprising that the energetic abnormalities associated with heart failure and arrhythmic risk also differ. Nevertheless, we believe this is the first report to relate a cardiac energetic abnormality, namely the depletion of ATP, important for maintaining ionic balance, to subsequent life-threatening clinical ventricular arrhythmias. Although mortality in patients with heart failure with a reduced ejection fraction was linked to a low myocardial PCr/ATP more than 2 decades ago (8), that study did not investigate arrhythmias or ICD firings. In the current study, when we used the same PCr/ATP criterion of less than 1.6 reported in the early work (8), we found that low PCr/ATP did not predict an increased arrhythmic risk. In fact, we observed a trend for low PCr/ATP to predict better, not worse, arrhythmic outcomes (Figure 2, G and H). This is because low ATP is important and appears in the denominator of the PCr/ATP ratio. The prior study of heart failure (33) and our current arrhythmic findings demonstrated that the cardiac energetic predictors of heart failure outcomes (CK flux) differed from those of arrhythmic risk (ATP levels). They also highlight that contemporary ^31^P MRS studies in patients that rely solely on PCr/ATP or assume constant ATP concentrations would underestimate the extent of metabolic abnormalities and potentially miss ATP depletion herein linked to life-threatening arrhythmic risk.

It should be acknowledged that our study was observational with a long enrollment phase and that ICD programming parameters were not prescriptive but determined by the implanting clinicians, as done in other contemporary ICD trials (35). Nevertheless, appropriate ICD firings were adjudicated by electrophysiologists as previously reported (15), and the appropriate ICD firing rates in our PROSe-ICD population were similar to those of contemporary trials where ICD programming was prescribed to minimize bias by nonsustained arrhythmias (3, 36). Although inappropriate ICD firing rates have declined over time in the PROSe-ICD and other ICD cohorts, likely influenced by published studies promoting changes in device programing (31), appropriate ICD firing rates have not declined (Supplemental Table 4 in ref. 36). Taken together, these observations suggest that the combination of clinically indicated ICD programming and blinded expert adjudication of appropriate ICD firings for ventricular tachycardia or ventricular fibrillation here reflect clinically relevant, life-threatening arrhythmias, the primary outcome measure of the current study that was predicted by cardiac ATP depletion.

The size of the study population was modest, which may have limited the number of potential confounders that could be adjusted for in MEA. Nevertheless, the population was large enough to detect significant associations of low myocardial ATP with subsequent life-threatening arrhythmias in multiple analyses, and many potential confounders were explored (Supplemental Table 2). Although SCD risk evolves over many years, it was not possible to repeat measures of myocardial energetics and ATP over time in these patients because once implanted, the ICDs create MRS artifacts, and an ICD is generally considered a contraindication to repeat ^31^P MRS studies, especially at 3 T. In addition, our follow-up data indicate that low cardiac ATP at baseline did not predict subsequent changes in NYHA class, LVEF, or NT-proBNP over approximately 2–3 years (Supplemental Figure 2). The limited spatial resolution and depth of interrogation of these older MRS studies may not fully characterize the spatial heterogeneity in energetics present throughout the heart (especially with prior infarction; ref. 37), but they did detect a significant association with human arrhythmogenesis. It is possible that newer, more sensitive metabolic MRS approaches offering improved spatial resolution may improve the detection of arrhythmia-relevant metabolic abnormalities in the future (38, 39). Finally, the cutoff values for normal myocardial energetic parameters were determined from previously published healthy controls because no healthy individuals qualified for a primary prevention ICD or were enrolled in PROSe-ICD. Nevertheless, identical acquisition and analysis protocols and MR scanners were used for healthy individuals and PROSe-ICD patients, and the studies were conducted contemporaneously.

In summary, these data demonstrate a significant link between cardiac ATP depletion detected noninvasively and life-threatening ventricular arrhythmogenesis in patients with reduced LVEF. These findings support testing of metabolic strategies that limit ATP loss as a treatment for or to prevent life-threatening cardiac arrhythmias. They highlight the potential of noninvasive ^31^P MRS as a complementary risk stratification tool in identifying individuals who would most benefit from ICDs for the primary prevention of SCD.

## Methods

### Study population.

This study reports the findings of the prospective metabolic substudy of the PROSe-ICD study relating cardiac high-energy metabolism to subsequent SCD risk in patients receiving primary prevention ICDs. A detailed description of the PROSe-ICD study design was previously published (15). Briefly, patients with no history of significant ventricular arrhythmias or SCD but who were scheduled to undergo primary prevention ICD implantation between November 2003 and December 2010 at the Johns Hopkins Medical Institutions were enrolled in the prospective study (ClinicalTrials.gov NCT00181233). All study participants underwent clinical assessment for LVEF 35% or less by echocardiography, nuclear scintigraphy, or ventriculography and a history of either ischemic or nonischemic cardiomyopathy. The decision for ICD insertion was clinically based on established guidelines (40). Participants with other indications for ICD placement (e.g., sustained ventricular arrhythmias, cardiac arrest, syncope) were not enrolled in this study. Participants were also excluded from the study if they had any contraindications to CMR (e.g., existing cardiac device, claustrophobia); had acute decompensated heart failure; had acute myocarditis or sarcoidosis or infiltrative disorders, such as amyloidosis or hemochromatosis; had congenital heart disease; or had hypertrophic cardiomyopathy. Participants with renal insufficiency (creatinine clearance < 30 mL/min after July 2006 or < 60 mL/min after February 2007) were also excluded from the study. Although the parent PROSe-ICD study recruited from several centers, this substudy recruited only patients receiving ICDs at Johns Hopkins Hospital. Because the MRS/MRI study had to be completed before clinically indicated ICD implantation, recruitment was primarily limited to individuals willing to undergo MRI/MRS evaluation when the scanner was available, within the short time window prior to ICD implantation. Supplemental Table 1 compares major demographic and clinical characteristics between this subcohort of PROSe-ICD and the parent PROSe-ICD group (as well as MADIT-II for comparison).

### ^31^P MRS protocol.

Resting cardiac ^31^P MRS data were collected on either a 1.5 T (General Electric Healthcare Technologies) or a 3.0 T (Achieva, Philips Healthcare) MRI/spectroscopy system shortly before ICD insertion. Spectra were acquired similarly in both ischemic and nonischemic participants lying prone on the MR table with the approximate location of the heart centered over a ^31^P receive/^31^P transmit surface coil set, as previously reported (7, 9, 37). All acquisition methods at 1.5 T and 3.0 T have been previously described and shown to give comparable results (7, 41–44). After the patient’s CMR spectroscopy examination, the final 2 steps were repeated, fully relaxed (repetition time [TR] = 4 seconds for ^1^H; TR = 8 seconds for ^31^P MRS), on a phosphate reference phantom to calibrate the ratio of phosphate to proton signal for accurately determining cardiac metabolite concentrations (41).

### Calculation of ΔG_~ATP_.

The ΔG_~ATP_ was determined via a previously used method (45):

where *ΔG_0_* is the standard free energy change, *R* is the universal gas constant, *T* is the absolute temperature, and [*Pi*] is the inorganic phosphate concentration. The cytosolic adenosine diphosphate (ADP) concentration was calculated according to , assuming an equilibrium constant of *K_eq_* = 1.66 × 10^9^ (46). [Cr], the unphosphorylated creatine, was calculated as the difference between total creatine and PCr, where [PCr] was directly measured by ^31^P MRS in these patients and total creatine taken from the literature (47). Because Pi is often difficult to detect in human cardiac ^31^P MR spectra given its low concentration and overlapping resonance of blood diphosphoglycerate (2,3-DPG), we conservatively assumed [*Pi*] = 1 mM for both the normal ATP and low ATP groups.

### CMR imaging protocol.

A CMR imaging protocol was performed on a separate visit, typically within 24 hours of the ^31^P MRS scan using either a GE 1.5 T (Signa CV/I, General Electric Healthcare Technologies) or a Siemens 1.5 T (Avanto) system. The details of the CMR imaging protocol were previously reported elsewhere (48, 49). This examination incorporated a series of left ventricular short-axis steady-state free precession cine images (TR = 3.8 ms; echo time = 1.6 ms; α = 45°; field of view = 36 to 40 cm; 8 mm slices; 256 × 160 points; 40 ms temporal resolution) for assessment of LVEF.

### ^31^P MRS analysis.

The ^31^P MRS analysis was performed while blinded to arrhythmic and clinical outcomes. Peak areas were first fitted in the frequency domain using either “CSX” or “circle fit” methods, as described previously (42, 50). The latter method fits a circle to each peak in a complex 3D plane, which reduces problems with phasing and baseline correction and yields results indistinguishable from the CSX method with an expert operator. ATP and PCr concentrations were then quantified after correcting for coil loading, relaxation, heart motion artifact, tissue volume, ATP in the ventricular luminal blood, and coil sensitivity variations within voxels using a MATLAB-based interface, as previously described (42). ATP concentration was quantified from the γ-ATP resonance peak. Finally, CK flux was calculated as the product of the CK forward rate constant (k_f_) and PCr and expressed as mmol/kg/s, where k_f_ was determined from the pseudo-first-order rate-constant of the CK reaction, measured using the four-angle saturation transfer method at 1.5 T (7, 44) and the triple-repetition time saturation transfer method at 3 T (43) with corrections for spillover irradiation (51, 52).

### CMR imaging analysis.

CMR images were analyzed using custom research software (Cinetool), the details of which were previously described elsewhere (53). Left ventricular mass, volumes, and ejection fraction were quantified using the method of disks (54).

### Clinical follow-up.

Patients were prospectively followed through clinic visits, study visits, telephone calls, and medical record review at 3- to 6-month intervals after ICD placement. ICD therapies were programmed at the discretion of the implanting electrophysiologist. An independent committee of board-certified electrophysiologists adjudicated all ICD firings and deaths while blinded to all clinical, biomarker, CMR, and ^31^P MRS data.

The primary endpoint was defined as the time to first occurrence of either (a) appropriate ICD firing for sustained ventricular tachycardia above the programmed ICD rate cutoff (generally 180 beats/min) or ventricular fibrillation or (b) cardiac death. Data were also analyzed separately for appropriate ICD firings alone (not including cardiac death). All other reasons for ICD firings were considered inappropriate and were not considered in the analyses. Deaths were classified according to the most proximate cause after review of ICD interrogations, medical records, death certificates, autopsy reports, and eyewitness accounts. Only deaths where cardiac involvement was the primary cause were considered as an event for the current investigation. The MEA used the occurrence of multiple events defined by the same criteria. Patients were censored at the time of heart transplant, left ventricular assist device placement, noncardiac death, or last contact.

### Statistics.

All statistical analyses were performed using either GraphPad Prism or SAS. Normal distributions were assessed by the Kolmogorov-Smirnov test. Continuous variables are reported as mean ± SD when normally distributed or as median with IQR when not. Categorical variables are reported as proportions. Survival curves were constructed according to the Kaplan-Meier method and compared the event-free survival of 2 dichotomous groups of low and normal high-energy phosphate concentrations as cutoff points. Low high-energy phosphates were defined as 2 standard deviations below the grouped mean of healthy individuals previously reported by our group (ATP: <3.4 μmol/g; PCr: <7.6 μmol/g; CK flux: <1.3 μmol/g/s) (7, 9). Event-free survival was compared between the 2 groups using the log-rank test. The marginal approach of Wei, Lin, and Weissfeld (WLW) was implemented in SAS software and used to perform MEA of time to a composite event, defined as appropriate ICD firing or cardiac death as well as for appropriate ICD firings alone. The WLW approach fits a Cox proportional hazards model to each component time to event and makes statistical inference of the regression parameters based on a robust “information sandwich”–type estimate of the standard error to accommodate within-subject correlations between events (55). MEA was performed with each high-energy phosphate variable (ATP, PCr, and CK flux) appearing in separate models. We controlled for the potentially confounding effect of low LVEF, which is known to be associated with SCD and ICD events (53, 56). Moreover, the MEA included other conventional risk factors previously associated with increased SCD risk (age, race, and sex). Variables with significantly elevated hazard ratios after controlling for these factors highlight their independent predictive value (55). A *P* value less than 0.05 was considered statistically significant.

### Study approval.

The study protocol was approved by the Johns Hopkins Hospital IRB, and all study participants provided written informed consent prior to participation in the study.

## Author contributions

RGW, GFT, and GG designed the study; RGW, GFT, and KCW obtained the funding; AMS and RGW recruited participants; MS and PAB performed the ^31^P MRS studies and analyzed those data; KCW analyzed the CMR data; KCW and GFT were responsible for follow-up studies; TJS combined the data and prepared the figures and tables; ACW and MEA performed the modeling analyses; SL performed statistical analysis; TJS, SL, PAB, GG, and RGW drafted the manuscript.

## Supplementary Material

Supplemental data

ICMJE disclosure forms

## Figures and Tables

**Table 3:**
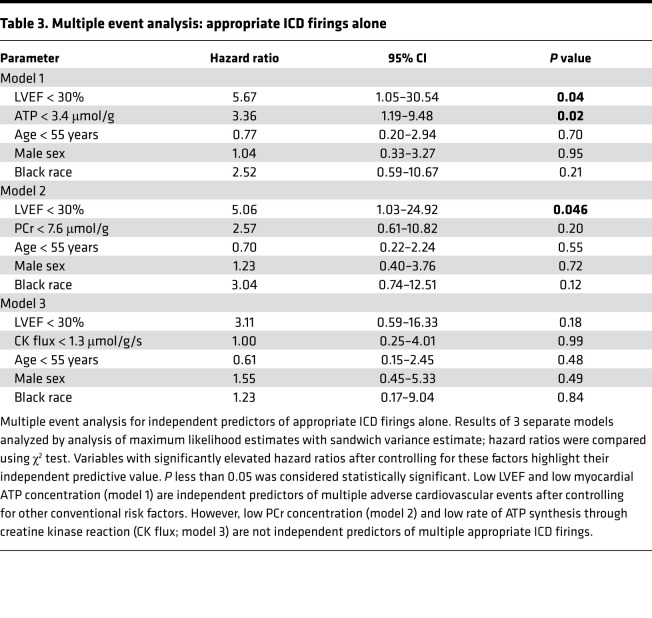
Multiple event analysis: appropriate ICD firings alone

**Table 2:**
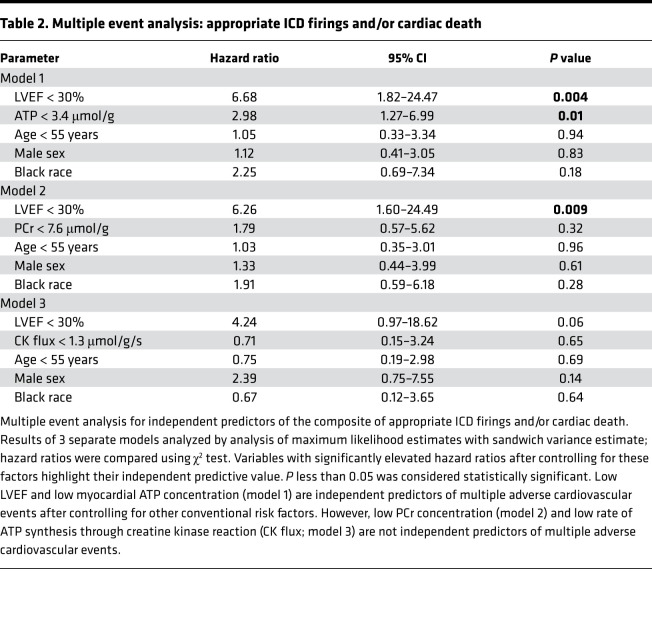
Multiple event analysis: appropriate ICD firings and/or cardiac death

**Table 1:**
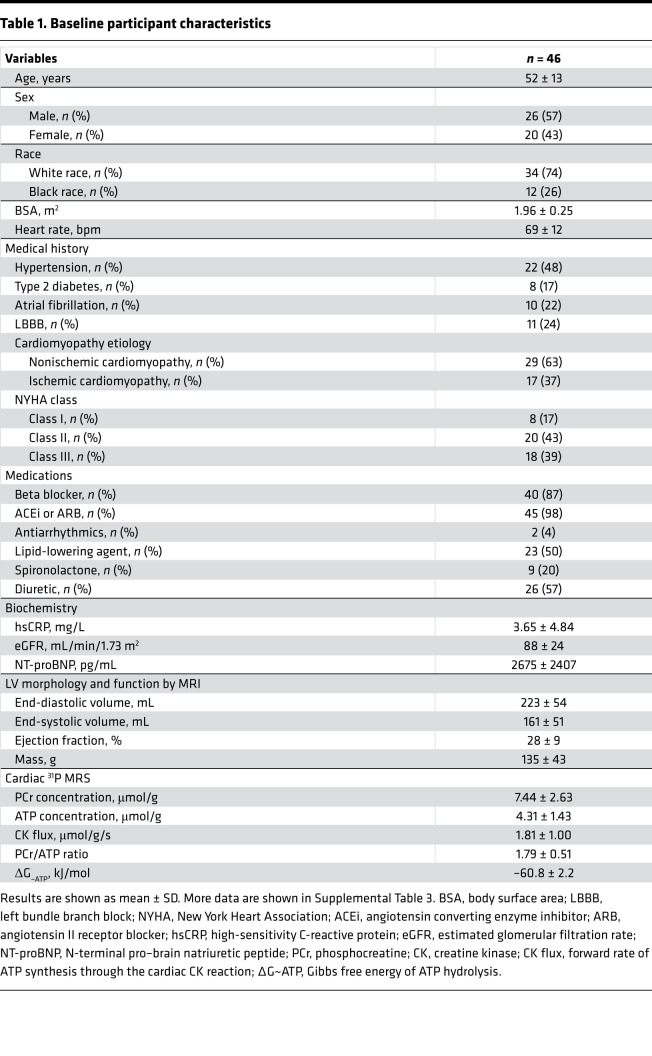
Baseline participant characteristics

## Version 1 05/17/2022

In-Press Preview

## Version 2 06/22/2022

Electronic publication
