# Supplementary material for: Myocardial ATP depletion detected noninvasively predicts sudden cardiac death risk in patients with heart failure
Source: JCI Insight. 2022 Jun 22;7(12):e157557. doi: 10.1172/jci.insight.157557 (PMC9309047; doi:10.1172/jci.insight.157557)
Supplement: Supplemental data [file jciinsight-7-157557-s152.pdf]

Data Supplement:

Supplement Table 1: Demographics of this PROSE-ICD cohort compared to that of the parent PROSE-ICD cohort and published MADIT-II populations (1, 2). Note that left ventricular ejection fraction reported here in PROSE-ICD was that at the time of study enrollment and based on available clinically-indicated echocardiographic, nuclear, CT, catheterization or MRI findings.

	PROSE-ICD Current Cohort (n = 46)	PROSE-ICD Entire Cohort (n = 1177)[1]	MADIT-II (n = 742)[2]
Age, years	52 ± 13	61 ± 13	64 ± 10
Male Sex, %	57	73	84
Non-White Race, %	26	43	0
Smoking History, %	52	67	80
History of Diabetes, %	17	35	33
History of Hypertension, %	48	63	53
LV Ejection Fraction, %	23 ± 7	23 ± 8	23 ± 5
NYHA Class			
Class I, %	17	17	35
Class II, %	43	43	35
Class III, %	39	38	25
Class IV, %	0	1	5
Ischemic Cardiomyopathy, %	37	54	100

[1] – from Cheng et al. (2013) J Am Heart Assoc (1).

[2] – from Moss et al. (2002) N Engl J Med (2).

Supplement Table 2: Demographic, co-morbidity, biomarker, MRI, MRS, electrocardiographic and electrophysiology data for study participants split by low and normal ATP.

	ATP <3.4µmol/g (n = 14)	ATP ≥3.4µmol/g (n=32)	P-Value
**Demographics**			
Age (years)	53 ± 13	52 ± 14	0.90
BSA (m^2)	1.92 ± 0.29	1.98 ± 0.24	0.45
Sex			
Male Sex, n(%)	7 (50)	19 (59)	0.56
Female Sex, n(%)	7 (50)	13 (41)	0.56
Race			
White Race, n(%)	11 (79)	23 (72)	0.63
Black Race, n(%)	3 (21)	9 (28)	0.63
NYHA Class, n(%)			0.21
I	1 (7)	7 (22)	0.23
II	5 (36)	15 (47)	0.48
III	8 (57)	10 (31)	0.098
**Medical History**			
History of Hypertension, n(%)	8 (57)	14 (44)	0.40
History of Diabetes, n(%)	5 (36)	3 (9)	0.030
Smoking History, n(%)	8 (57)	16 (50)	0.66
Hypercholestaremia, n(%)	7 (50)	14 (44)	0.70
Atrial Fibrillation, n(%)	5 (36)	5 (16)	0.13
Left-Bundle Branch Block, n(%)	5 (36)	6 (19)	0.21
Ischemic Cardiomyopathy, n(%)	8 (57)	9 (28)	0.061
Duration of Cardiomyopathy (years)	1.7 (0.2-7.9)	2.4 (0.7-9.8)	0.43
**Medications**			
Aspirin, n(%)	12 (86)	18 (56)	0.054
β-Blockers, n(%)	13 (93)	27 (84)	0.43
ACEi or ARB, n(%)	14 (100)	31 (97)	0.50
Anti-Arrhythmics, n(%)	1 (7)	1 (3)	0.54
Lipid Lowering, n(%)	9 (64)	14 (44)	0.20
Spironolactone, n(%)	3 (21)	6 (19)	0.83
Diuretic, n(%)	10 (71)	16 (50)	0.18
Hydralazine, n(%)	1 (7)	0 (0)	0.054
Digoxin, n(%)	3 (21)	6 (19)	0.83
Aldosterone Inhibitor, n(%)	3 (21)	6 (19)	0.87
**^{31}P MRS**			
PCr Concentration (µmol/g)	5.2 (5.0-5.7)	7.6 (6.0-10.1)	0.0002
ATP Concentration (µmol/g)	2.7 ± 0.5	5.0 ± 1.1	By Design
CK Flux (µmol/g/s)	1.42 ± 0.54	1.99 ± 1.12	0.092
k used (/s)	0.26 ± 0.12	0.25 ± 0.13	0.81
PCr/ATP Ratio	1.92 (1.77-2.27)	1.62 (1.38-1.79)	0.0007
$\Delta G_{\sim ATP}$, kJ/mol	-59.2 (-59.6- -59.1)	-60.9 (-62.5- -59.8)	0.0002
**Biomarkers**			
hsCRP (mg/L)	2.76 (1.41-4.98)	1.86 (0.63-4.35)	0.28
eGFR (mL/min/1.73m^2)	87.3 ± 34.3	88.1 ± 18.3	0.92
TNF-α (pg/mL)	2369 (1999-4329)	2319 (1870-3311)	0.57

	hsIL-6 (pg/mL)	1.61 (1.06-3.00)	1.19 (0.62-2.71)	0.31
	NT-proBNP (pg/mL)	2295 (1708-6968)	1560 (1299-3130)	0.043
	Serum Na (mEq/L)	138 ± 3	139 ± 2	0.18
	Serum Cr (mg/dL)	0.9 (0.7-1.1)	0.9 (0.8-1.0)	0.99
	Serum K (mEq/L)	4.34 ± 0.33	4.19 ± 0.35	0.17
**MRI Measures**				
	LVEDV (mL)	231 ± 65	220 ± 49	0.51
	LVESV (mL)	157 (137-199)	142 (125-195)	0.49
	LVEF (%)	27 ± 10	29 ± 8	0.37
	LV Mass (g)	124 (109-188)	126 (111-140)	0.61
**Electrophysiology**				
	Inducible VT/VF, n(%)	6 (43)	12 (38)	0.89
	Monomorphic VT at EPS, n(%)	5 (36)	6 (19)	0.27
	VT Cycle Length at EPS	255 ± 57	238 ± 33	0.53
	12-lead QRS (ms)	122 (91-146)	103 (96-128)	0.45
	12-lead QTc (ms)	459 (430-494)	438 (410-472)	0.078
	SAECG – HF QRS (ms)	116 (86-149)	100 (84-142)	0.47
	SAECG – RMS last 40ms (mV)	24 (14-37)	20 (12-36)	0.97
	SAECG – Duration under 40mV (ms)	37 (22-56)	32 (22-56)	0.79
	SAECG – HF Noise (mV)	0.55 (0.29-0.87)	0.36 (0.27-0.51)	0.097
	HRV – Max R-R (ms)	1054 (832-1220)	1180 (1034-1450)	0.094
	HRV – Min R-R (ms)	654 (452-774)	708 (490-808)	0.54
	HRV – Average R-R (ms)	893 ± 151	929 ± 163	0.49
	HRV – SDNN (ms)	33 (23-99)	48 (32-127)	0.24
	HRV – RMSSD (ms)	26 (11-119)	50 (25-178)	0.16
	HRV – HRV Triangle Index	10 (8-12)	12 (8-22)	0.15
	HRV – ULF	56 (1-163)	8 (2-20)	0.42
	HRV – VLF	200 (86-362)	111 (52-198)	0.095
	HRV – LF	125 (65-183)	117 (85-167)	0.96
	HRV – HF	126 ± 106	178 ± 89	0.10
	HRV – LF/HF	0.88 (0.61-1.69)	0.65 (0.45-1.17)	0.18
	QTv – Max QT (ms)	524 ± 67	539 ± 94	0.63
	QTv – Min QT (ms)	328 (277-385)	336 (281-374)	0.92
	QTv – Average QT (ms)	423 (392-456)	417 (381-443)	0.68
	QTv – SDNN (ms)	29 ± 19	30 ± 18	0.92
	QTv – RMSSD (ms)	39 ± 27	36 ± 22	0.73
	QTv – QTV Triangle Index	3.54 (2.82-7.02)	5.23 (2.95-7.68)	0.57
	QTv – ULF	22 (1-46)	19 (9-37)	0.99
	QTv – VLF	50 (34-67)	83 (54-101)	0.022
	QTv – LF	122 (102-169)	114 (100-137)	0.45
	QTv – HF	181 ± 115	223 ± 135	0.34
	QTv – LPF/HPF	0.61 (0.47-0.91)	0.45 (0.34-0.71)	0.38

Categorical data are presented as n(%) and were analyzed using the Chi-squared test. Continuous data are presented as mean ± SD if normally distributed and were analyzed using Student's t-test, if normal distribution was not confirmed data are presented as median (IQR) and were analyzed using the Mann-Whitney U test.

Supplement Table 3: Complete list of baseline participant characteristics.

Variables	*n* = 46
**Anthropometrics**	
Age, years	52 ± 13
Sex	
Male, n (%)	26 (57)
Female, n (%)	20 (43)
Race	
White Race, n (%)	34 (74)
Black Race, n (%)	12 (26)
BSA, m^2	1.96 ± 0.25
Heart Rate, bpm	69 ± 12
**Medical History**	
Hypertension, n (%)	22 (48)
Type-2 Diabetes, n (%)	8 (17)
Atrial Fibrillation, n (%)	10 (22)
Hypercholesterolemia, n (%)	21 (46)
Smoking, n (%)	24 (52)
LBBB, n (%)	11 (24)
Cardiomyopathy Etiology	
Non-ischemic Cardiomyopathy, n (%)	29 (63)
Ischemic Cardiomyopathy, n (%)	17 (37)
NYHA Class	
Class I, n (%)	8 (17)
Class II, n (%)	20 (43)
Class III, n (%)	18 (39)
**Medications**	
β-blocker, n (%)	40 (87)
ACEi or ARB, n (%)	45 (98)
Anti-arrhythmics, n (%)	2 (4)
Lipid Lowering Agent, n (%)	23 (50)
Aspirin, n (%)	30 (65)
Spironolactone, n (%)	9 (20)
Diuretic, n (%)	26 (57)
Hydralazine, n (%)	1 (2)
Digoxin, n (%)	9 (20)
Aldosterone Inhibitor, n (%)	9 (20)
**Biochemistry**	
hsCRP, mg/L	3.65 ± 4.84
eGFR, mL/min/1.73m^2	88 ± 24
TNF-α Receptor-II, pg/mL	2854 ± 1315
hsIL-6, pg/mL	3.46 ± 6.91
NT-proBNP, pg/mL	2675 ± 2407
Serum Sodium, mEq/L	139 ± 2
Serum Creatinine, mg/dL	0.9 ± 0.3
Serum Potassium, mEq/L	4.2 ± 0.3

LV Morphology and Function by MRI	
End-diastolic Volume, mL	223 ± 54
End-systolic Volume, mL	161 ± 51
Ejection Fraction, %	28 ± 9
Mass, g	135 ± 43
**Cardiac ^{31}P MRS**	
PCr Concentration, µmol/g	7.44 ± 2.63
ATP Concentration, µmol/g	4.31 ± 1.43
CK flux, µmol/g/s	1.81 ± 1.00
PCr/ATP Ratio	1.79 ± 0.51
$\Delta G_{\sim ATP}$, kJ/mol	-60.8 ± 2.2

BSA – body surface area; LBBB – left bundle branch block; NYHA – New York Heart Association; ACEi – angiotensin converting enzyme inhibitor; ARB – angiotensin-II receptor blocker; hsCRP – high sensitivity C-reactive protein; eGFR – estimated glomerular filtration rate; TNF-α – tumor necrosis factor-α; hsIL-6 – high sensitivity interleukin-6; NT-proBNP – N-terminal pro-brain natriuretic peptide; PCr - phosphocreatine; ATP – adenosine triphosphate; CK – creatine kinase; CK flux- forward rate of ATP synthesis through the cardiac CK reaction; $\Delta G_{\sim ATP}$ – Gibbs free energy of ATP hydrolysis. Results are shown as mean ± SD.

Supplement Figure 1: Relationship between myocardial [ATP] and left ventricular ejection fraction (LVEF; Pearson's correlation test).

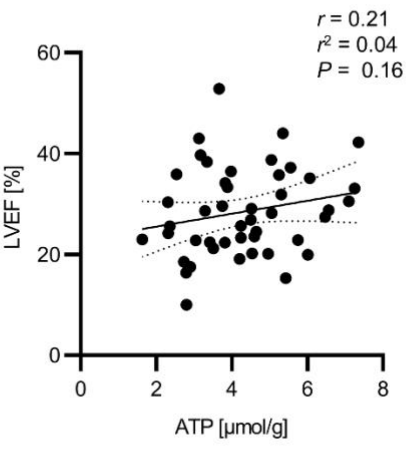

Supplement Figure 2: During the first 3 years of follow-up post-ICD implantation, no group differences were observed between the low and normal myocardial ATP groups in terms of change in NYHA Class, left ventricular ejection fraction (LVEF) and NT-proBNP. This is consistent with the hypothesis that the ability of low myocardial ATP to predict sudden cardiac death risk is not simply a reflection of low ATP as a predictor of heart failure progression. Data represent mean ± SD and group differences were assessed by Student's t-test.

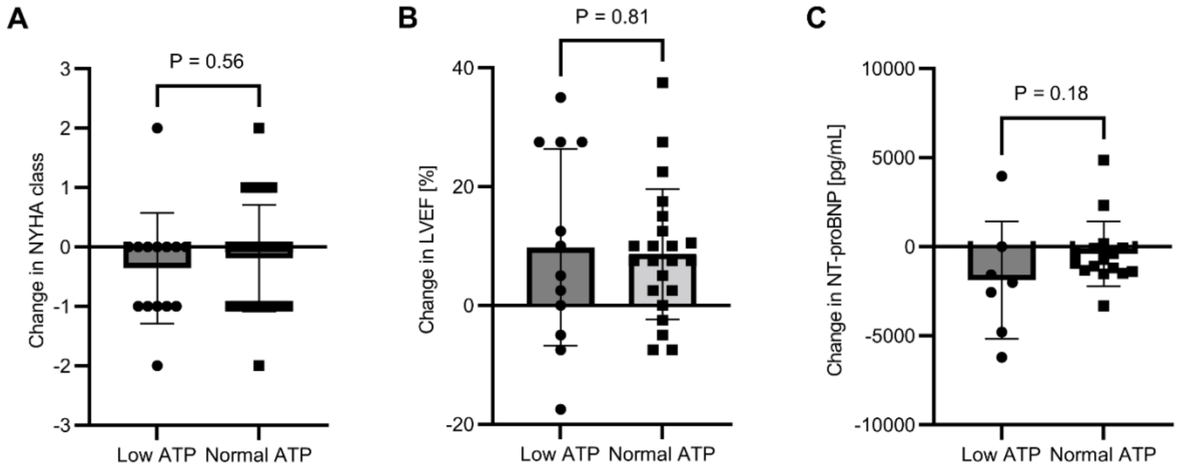

Supplement Figure 3: Results of computational modeling for the impact of ATP depletion on a simulated action potential (AP, panel A) and SERCA activity (panel B, "Jup") for normal ATP (solid line) and low ATP conditions (dotted line). The computational model employed (3) integrates mitochondrial bioenergetics and electrophysiology in the cardiac myocyte. The general, membrane and specific ion current, ion pump, and energetic parameters were assumed to be similar for normal ATP and low ATP conditions. The high-energy phosphate parameters were derived from the patients studied here and appear in Supplemental Table 2). Details of the model appear at the end of this Supplement and the code is provided in a separate file. The findings suggest that the "low ATP" energetic profile per se, prolongs the action potential, reduces SERCA calcium handling, as compared to "normal ATP".

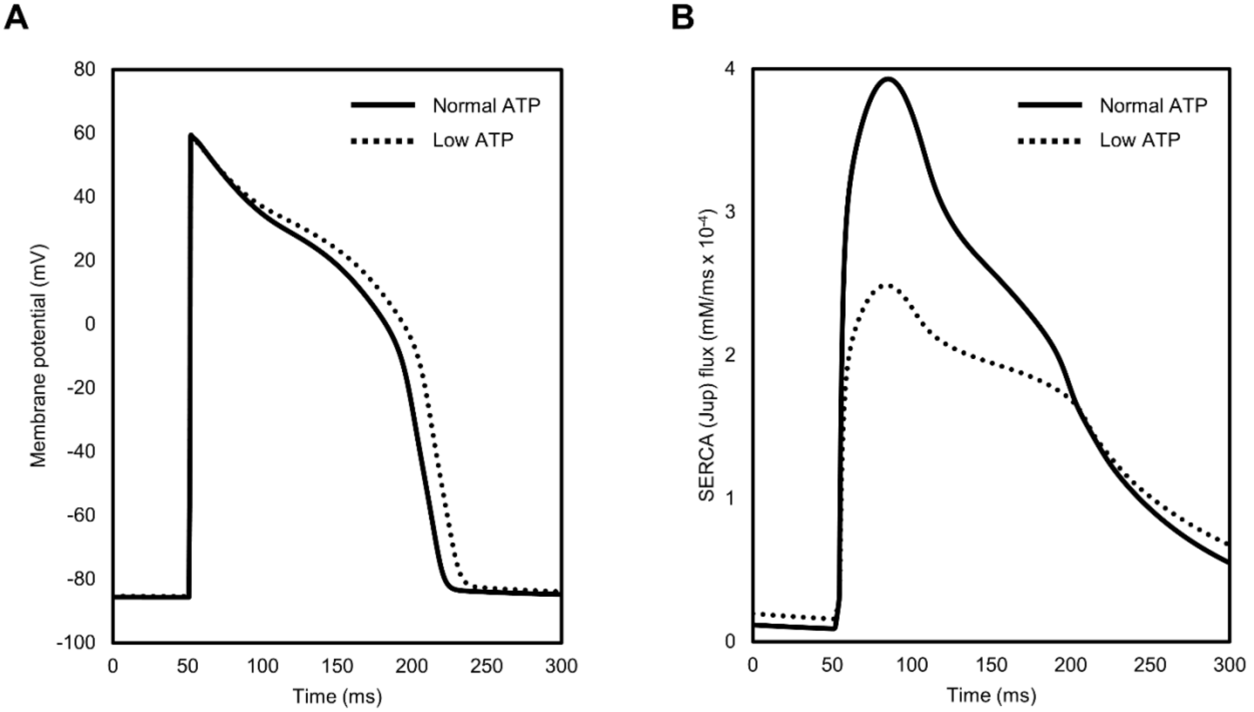

Reference List

1. Cheng A, Dalal D, Butcher B, Norgard S, Zhang Y, Dickfeld T, et al. Prospective observational study of implantable cardioverter-defibrillators in primary prevention of sudden cardiac death: study design and cohort description. *J Am Heart Assoc.* 2013;2(1):e000083.
2. Moss AJ, Zareba W, Hall WJ, Klein H, Wilber DJ, Cannom DS, et al. Prophylactic implantation of a defibrillator in patients with myocardial infarction and reduced ejection fraction. *N Engl J Med.* 2002;346(12):877-83.
3. Cortassa S, Aon MA, O'Rourke B, Jacques R, Tseng HJ, Marban E, et al. A computational model integrating electrophysiology, contraction, and mitochondrial bioenergetics in the ventricular myocyte. *Biophys J.* 2006;91(4):1564-89.
4. Vinnakota KC, and Bassingthwaighte JB. Myocardial density and composition: a basis for calculating intracellular metabolite concentrations. *Am J Physiol Heart Circ Physiol.* 2004;286(5):H1742-9.

Supplementary information for model equations and parameters

The computational simulation is based on excitation-contraction coupling/mitochondrial energetics (ECME) model of Cortassa et al (3). The ECME model contains 51 ordinary differential equations (ODEs) to describe the dynamic changes of ions and metabolites in guinea pig cardiac ventricular myocytes. Note that the main elements of this model were originally constrained using normal guinea pig data, because human data are often not available for many parameters and because guinea pig myocyte critical electrophysiologic and Ca^{++} handling parameters are similar to those in humans. The code was converted from CellML (S1.xml in Physiome Model Repository) to MATLAB in OpenCOR and ran in MATLAB2021b.

The code is available at https://gitlab.com/MitoModel/ecme_hf.

The parameters were directly taken from the ECME model. To simulate the conditions in this study of a normal ATP level and low ATP level, the cytosolic ATP and creatine phosphate levels were clamped to the constant levels as follows (derived from Supplement Table 2 above). Note that metabolite concentrations were converted from umol/g wet wt to mM with cytosolic volume taken as 0.725 ml/g of wet weight (4).

	PCr/ATP	$[ATP]_{cyto}$ (mM)	$[PCr]_{cyto}$ (mM)	Total Creatine ([Cr]+[PCr]) (mM)
HF with normal ATP	1.62	6.9	11.178	22.178
HF with low ATP	1.97	3.7	7.289	22.189

, and set $\frac{dPCr_{cyto}}{dt} = 0, \frac{dATP_{cyto}}{dt} = 0.$

I. General parameters

Parameter	Value	Unit	Desc.
F	96485	C/mol	Faraday constant
C_m	1.0	µF cm^{-2}	Membrane capacitance
A_{cap}	1.534 10^{-4}	cm^2	Capacitative cell surface area
V_{myo}	25.84	pL	Cytosolic volume
V_{mito}	15.89	pL	Mitochondrial volume
V_{NSR}	1.4	pL	NSR volume
V_{JSR}	0.16	pL	JSR volume
V_{SS}	0.495 10^{-3}	pL	SS volume
$[K^+]_o$	5.4	mM	Extracellular K^+ concentration
$[Na^+]_o$	140.0	mM	Extracellular Na^+ concentration
$[Ca^{2+}]_o$	2.0	mM	Extracellular Ca^{2+} concentration
C_m	1.0	µF cm^{-2}	Membrane capacitance

II. Sarcoplasmic ion currents

Time-dependent delayed rectifier potassium current (IK)

$$
\begin{aligned}
I_K &= \bar{G}_K X_1 X_K^2 (V - E_K) \\
E_K &= E_N\left([K^+]_o + P_{Na,K}[Na^+]_o, [K^+]_i + P_{Na,K}[Na^+]_i, z_K\right) \\
\bar{G}_K &= 0.282\sqrt{[K^+]_o/5.4} \\
X_1 &= \left(1 + e^{(V_m - 40)/40}\right)^{-1} \\
\frac{dX_k}{dt} &= \alpha_X - X_k(\alpha_X + \beta_X) \\
\alpha_X &= 7.19 \cdot 10^{-5} \frac{V_m + 30}{1 - e^{-0.148(V_m+30)}} \\
\beta_X &= 1.31 \cdot 10^{-4} \frac{V_m + 30}{e^{0.0687(V_m+30)} - 1}
\end{aligned}
$$

Time-independent potassium current (IK1)

$$\Delta V = V_m - E_{K1}$$
$$I_{K1} = \bar{G}_{K1} K_{1\infty} \Delta V$$
$$E_{K1} = E_N(K^+]_o, [K^+]_i, 1)$$
$$\bar{G}_{K1} = 0.748\sqrt{[K^+]_o/5.4}$$
$$K_{1\infty} = \frac{\alpha_{K_1}}{\alpha_{K_1} + \beta_{K_1}}$$
$$\alpha_{K_1} = \frac{1.02}{1 + e^{0.2385(\Delta V - 59.215)}}$$
$$\beta_{K_1} = \frac{0.4912 e^{0.28032(\Delta V + 5.476)} + e^{0.06175(\Delta V - 594.31)}}{1 + e^{-0.5143(\Delta V + 4.753)}}$$

Plateau potassium current (IKp)

$$E_{Kp} = E_N([K^+]_o, [K^+]_i, z_K)$$
$$I_{Kp} = \frac{\bar{G}_{Kp}\left(V - E_{Kp}\right)}{1 + e^{(7.488 - V_m)/5.98}}$$

Fast Na current (INa)

$$I_{Na} = \bar{G}_{Na} m^3 hj (V_m - E_{Na})$$
$$E_{Na} = E_N([Na^+]_o, [Na^+]_i, 1)$$
$$\frac{dm_{Na}}{dt} = \alpha_m - m_{Na}(\alpha_m + \beta_m)$$
$$\frac{dh_{Na}}{dt} = \alpha_h - h_{Na}(\alpha_h + \beta_h)$$
$$\frac{dj_{Na}}{dt} = \alpha_j - m_{Na}\left(\alpha_j + \beta_j\right)$$
$$\alpha_m = 0.32 \frac{V + 47.13}{1 - e^{-0.1(V_m + 47.13)}}$$
$$\beta_m = 0.08 e^{-V_m/11}$$

$$For\ V \geq -40mV$$
$$\alpha_h = \alpha_j = 0$$
$$\beta_h = \left(0.13\left(1 + e^{-(V_m + 10.66)/11.1}\right)\right)^{-1}$$
$$\beta_j = 0.3 \frac{e^{-2.535 \cdot 10^{-7} V_m}}{1 + e^{-0.1(V_m + 32)}}$$

$$For\ V < -40mV$$
$$\alpha_h = 0.135 e^{-(V_m + 80)/6.8}$$
$$\alpha_j = (-127140 e^{0.2444 V_m} - 3.474 \cdot 10^{-5} e^{-0.04391 V_m}) \frac{V_m + 37.78}{1 + e^{0.311(V_m + 79.23)}}$$
$$\beta_h = 3.56 e^{0.079 V_m} + 3.1 \cdot 10^5 e^{0.35 V_m}$$
$$\beta_j = \frac{0.1212 e^{-0.01052 V_m}}{1 + e^{-0.1378(V_m + 40.14)}}$$

Sodium-calcium exchanger current (INaCa)

$$
\begin{aligned}
I_{NaCa} &= k_{NaCa} \cdot f_{Nao} \cdot f_{Cao} \frac{exp(V_m/V_T)\phi_{Na}^3 - \phi_{Ca}}{exp\big((1-\eta)V_m/V_T\big) + k_{sat}} \\
f_{Nao} &= Hill\big([Na^+]_o, K_{M,Na}^{NaCa}, 3\big) \\
f_{Cao} &= Hill\big([Ca^+]_o, K_{M,Ca}^{NaCa}, 1\big) \\
\phi_{Na} &= [Na^+]_i/[Na^+]_o \\
\phi_{Ca} &= [Ca^+]_i/[Ca^+]_o
\end{aligned}
$$

Background calcium ($\boldsymbol{I_{Ca,b}}$) and sodium currents ($\boldsymbol{I_{Na,b}}$)

$$
\begin{aligned}
I_{Ca,b} &= \bar{G}_{Ca,b}(V - E_{Ca}) \\
E_{Ca} &= E_N([Ca^{2+}]_o, [Ca^{2+}]_i, z_{Ca}) \\
I_{Na,b} &= \bar{G}_{Na,b}(V - E_{Na}) \\
E_{Na} &= E_N([Na^+]_o, [Na^+]_i, z_{Na})
\end{aligned}
$$

Non-specific calcium-activated current (InsCa)

$$
\begin{aligned}
f_{Ca} &= Hill([Ca^{2+}]_i,\ K_m^{nsCa},\ 3) \\
I_{nsNa} &= 0.75 \cdot f_{Ca} \cdot \Phi_{Na}(P_{nsNa}, z_{Na}, V_m, [Na^+]_i, [Na^+]_o) \\
I_{nsK} &= 0.75 \cdot f_{Ca} \cdot \Phi_K(P_{nsK}, z_K, V_m, [K^+]_i, [K^+]_o)
\end{aligned}
$$

Sodium-potassium ATPase current (INaK)

$$
\begin{aligned}
\sigma &= \frac{e^{[Na^+]_o/67.3} - 1}{7} \\
f_{NaK} &= \big(1 + 0.1245 exp(-0.1V_m/V_T) + 0.0365\sigma exp(-V_m/V_T)\big)^{-1} \\
f_{Na} &= Hill\big([Na^+]_i,\ K_{m,Na_i}, 1.5\big) \\
f_K &= Hill\big([K^+]_o,\ K_{m,K_o}, 1\big) \\
f_{ATP} &= Hill\big([ATP]_i \cdot Hill\big(K_{i,ADP}^{NaK}, [ADP]_i, 1\big),\ K_{M,ATP}^{NaK}, 1\big) \\
I_{NaK} &= \bar{I}_{NaK} \cdot f_{ATP} \cdot f_{Na} \cdot f_K \cdot f_{NaK}
\end{aligned}
$$

ODE for electrophysiology

$$
\begin{aligned}
\frac{d[Na^+]_i}{dt} &= -(I_{Na} + 3I_{NaCa} + 3I_{NaK})\frac{A_{cap}}{V_{myo}F} + (V_{NHE} - 3V_{NaCa})\frac{V_{mito}}{V_{myo}} \\
\frac{d[K^+]_i}{dt} &= -\big(I_{Ks} + I_{Kr} + I_{K1} + I_{Kp} + I_{Ca,K} - 2I_{NaK}\big)\frac{A_{cap}}{V_{myo}F} \\
\frac{dV_m}{dt} &= -\frac{1}{C_m}\big(I_{Na} + I_{CaL} + I_{Kr} + I_{Ks} + I_{K1} + I_{Kp} + I_{NaCa} + I_{NaK} + I_{pCa} + I_{Ca,b} + I_{K_{ATP}} + I_{stim}\big)
\end{aligned}
$$

Functions

Nernst potential

$$E_N(X_o, X_i, z) := \frac{RT}{Fz} ln\left(\frac{X_o}{X_i}\right) \approx \frac{26.7mV}{z} ln\left(\frac{X_o}{X_i}\right)$$

Hill function

$$Hill(x, k, n) := \frac{x^n}{x^n + k^n}$$

GHK current equation

$$\Phi_s(P_s, z_s, V_m, [S]_i, [S]_o) := P_s z_s^2 \frac{V_m F^2}{RT} \frac{[S]_i - [S]_o \exp(-z_s V_m F/RT)}{1 - \exp(-z_s V_m F/RT)}$$

Parameters

Symbol	Value	Units	Description
$\overline{G}_{Na}$	12.8	$mS \cdot cm^{-2}$	Maximal Na+ channel conductance
$\overline{G}_{Kp}$	0.00828	$mS \cdot cm^{-2}$	Maximal plateau K+ channel conductance
$\overline{G}_{K,0}$	0.282	$mS \cdot cm^{-2}$	IK conductance
$\overline{G}_{K1,0}$	0.748	$mS \cdot cm^{-2}$	IK1 conductance
P_{NaK}	0.01833		Na+ permeability ratio of K+ channel
K_{NaCa}	9000	$\mu A \cdot cm^{-2}$	NCX current
$K_{M,Na}^{NaCa}$	87.5	mM	Dissociation constant of sodium for NCX
$K_{M,Ca}^{NaCa}$	1.38	mM	Dissociation constant of calcium for NCX
K_{sat}^{NaCa}	0.1		NCX saturation factor at negative potentials
η^{NCX}	0.35		Voltage dependence of NCX
$P_{ns,Na}$	$1.75 \cdot 10^{-7}$	$cm\ s^{-1}$	Nonspecific channel current Na permeability
$P_{ns,K}$	0	$cm\ s^{-1}$	Nonspecific channel current K permeability
K_{ca}^{ns}	1.2	μM	Ca2+ half-saturation constant for nonspecific current
$\bar{G}_{Ca,b}$	0.003217	$mS \cdot cm^{-2}$	Maximum background current Ca2+ conductance
$\bar{G}_{Na,b}$	0.003217	$mS \cdot cm^{-2}$	Maximum background current Na+ conductance
$\bar{I}_{NaK}$	3.147	$\mu A \cdot cm^{-2}$	Maximum Na^+/K^+ pump current
K_{m,Na_i}	10	mM	Na+ half saturation for Na^+/K^+ pump
K_{m,K_o}	1.5	mM	K+ half saturation for Na^+/K^+ pump
$K_{NaK}^{1,ATP}$	8.0×10^{-3}	mM	ATP half saturation constant for Na^+/K^+ pump
$K_{NaK}^{i,ADP}$	0.1	mM	ADP inhibition constant for Na^+/K^+ pump

III. Cytosolic calcium dynamics

L-type Ca current (ICa & ICaK)

Common pool of subspace calcium model

$$
\begin{aligned}
\alpha &= 0.4e^{(V_m+2)/10} \\
\beta &= 0.4e^{-(V_m+2)/13} \\
\alpha' &= a\alpha \\
\beta' &= \beta/b \\
\gamma &= 0.1875[Ca^{2+}]_{ss} \\
C_0 &= 1 - C_0 - C_1 - C_2 - C_3 - C_4 - O - C_{Ca0} - C_{Ca1} - C_{Ca2} - C_{Ca3} - C_{Ca4} \\
v_{01} &= 4\alpha C_0 - \beta C_1 \\
v_{12} &= 3\alpha C_1 - 2\beta C_2 \\
v_{23} &= 2\alpha C_2 - 3\beta C_3 \\
v_{34} &= \alpha C_3 - 4\beta C_4 \\
v_{45} &= fC_4 - gO \\
v_{67} &= 4\alpha' C_{Ca0} - \beta' C_{Ca1} \\
v_{78} &= 3\alpha' C_{Ca1} - 2\beta' C_{Ca2} \\
v_{89} &= 2\alpha' C_{Ca2} - 3\beta' C_{Ca3} \\
v_{910} &= \alpha' C_{Ca3} - 4\beta' C_{Ca4} \\
v_{06} &= \gamma C_0 - \omega C_{Ca0} \\
v_{17} &= a\gamma C_1 - \omega C_{Ca1}/b \\
v_{28} &= a^2\gamma C_2 - \omega C_{Ca2}/b^2 \\
v_{39} &= a^3\gamma C_3 - \omega C_{Ca3}/b^3 \\
v_{410} &= a^4\gamma C_4 - \omega C_{Ca4}/b^4 \\
\frac{dC_0}{dt} &= -v_{01} - v_{06} \\
\frac{dC_1}{dt} &= v_{01} - v_{12} - v_{17} \\
\frac{dC_2}{dt} &= v_{12} - v_{23} - v_{28} \\
\frac{dC_3}{dt} &= v_{23} - v_{34} - v_{34} \\
\frac{dC_4}{dt} &= v_{34} - v_{45} - v_{410} \\
\frac{dO}{dt} &= v_{45} \\
\frac{dC_{Ca0}}{dt} &= v_{06} - v_{67} \\
\frac{dC_{Ca1}}{dt} &= v_{17} + v_{67} - v_{78} \\
\frac{dC_{Ca2}}{dt} &= v_{28} + v_{78} - v_{89} \\
\frac{dC_{Ca3}}{dt} &= v_{39} + v_{89} - v_{910}
\end{aligned}
$$

$$
\begin{aligned}
I_{Ca}^{max} &= \Phi_{Ca}(P_{Ca}, z_{Ca}, V_m, 0.001, 0.341[Ca^{2+}]_o)\\
I_{Ca} &= 6I_{Ca}^{max} \cdot y_{Ca} \cdot O\\
I_{Ca,K} &= y_{Ca} \cdot O \cdot \Phi_{Ca}(P_K, z_K, V_m, [K^+]_i, [K^+]_o)\\
P_K &= P_K^{max} \cdot Hill\left(I_{Ca}^{half}, I_{Ca}^{max}, 1\right)\\
y_\infty &= \frac{1}{1+e^{(V_m+55)/7.5}} + \frac{0.5}{1+e^{(-V_m+21)/6}}\\
\tau_y &= 20 + \frac{600}{1+e^{(V_m+30)/9.5}}\\
\frac{dy_{Ca}}{dt} &= \frac{y_\infty - y_{Ca}}{\tau_y}
\end{aligned}
$$

Parameter	Value	Units	Description
A	2		Mode transition parameter
B	2		Mode transition parameter
ω	10	s^{-1}	Mode transition parameter
f	300	s^{-1}	Transition rate into open state
g	2000	s^{-1}	Transition rate into open state
f'	0	s^{-1}	Transition rate into open state
g'	0	s^{-1}	Transition rate into open state
$P_{Ca}^{LCC}(P_{Ca})$	$1.24 \cdot 10^{-3}$	$cm\ s^{-1}$	L-type Ca2+ channel permeability to Ca2+
$P_K^{LCC}(P_K^{max})$	$1.11 \cdot 10^{-11}$	$cm\ s^{-1}$	L-type Ca2+ channel permeability to K+
$I_{Ca,half}$	-0.4583	$\mu A/cm^2$	ICa level that reduces equation Pk by half

Ryanodine receptor (calcium release, Jrel)

$$P_{C1} = 1 - P_{O1} - P_{O2} - P_{C2}$$

$$If\ [Ca^{2+}]_{ss} \geq [Ca^{2+}]_{ss}^{*}:$$
$$P_{O1} := (P_{O1} + P_{C1})Hill(k_a^{+}[Ca^{2+}]_{ss}^{n}, k_a^{-}, 1)$$
$$v_{o1c1} = 0$$
$$If\ [Ca^{2+}]_{ss} < [Ca^{2+}]_{ss}^{*}:$$
$$v_{o1c1} = -k_a^{-}P_{O1} + k_a^{+}[Ca^{2+}]_{ss}^{n}P_{C1}$$

$$v_{o1o2} = k_b^{+}[Ca^{2+}]_{ss}^{m}P_{O1} - k_b^{-}P_{O2}$$
$$v_{o1c2} = k_c^{+}P_{O1} - k_c^{-}P_{C2}$$
$$\frac{dP_{O1}}{dt} = -v_{o1c1} - v_{o1o2} - v_{o1c2}$$
$$\frac{dP_{O2}}{dt} = v_{o1o2}$$
$$\frac{dP_{C2}}{dt} = v_{o1c2}$$
$$J_{rel} = r_{ryr}(P_{O1} + P_{O2})\left([Ca^{2+}]_{JSR} - [Ca^{2+}]_{ss}\right)$$

Parameter	Value	Units	Description
v_1	3600	s^{-1}	RyR flux channel constant
n	4		Cooperativity parameter
m	3		Cooperativity parameter
k_a^{+}	$1.215\text{x}10^{13}$	s^{-1} mM^{-4}	RyR rate constant
k_a^{-}	576	s^{-1}	RyR rate constant
k_b^{+}	$4.05\text{x}10^{6}$	s^{-1} mM^{-3}	RyR rate constant
k_b^{-}	1930	s^{-1}	RyR rate constant
k_c^{+}	100	s^{-1}	RyR rate constant
k_c^{-}	0.8	s^{-1}	RyR rate constant

Plasma membrane calcium ATPase (PMCA) current (IpCa)

$$f_{ATP} = Hill\left([ATP]_i \cdot Hill\left(K_{i,ADP}^{PMCA}, [ADP]_i, 1\right), K_{M1,ATP}^{PMCA}, 1\right) + Hill\left([ATP]_i, K_{M2,ATP}^{PMCA}, 1\right)$$

$$f_{Ca} = Hill\left([Ca^{2+}]_i,\ K_{M,Ca}^{PMCA}, 1\right)$$

$$I_{pCa} = I_{max}^{PMCA} \cdot f_{Ca} \cdot f_{ATP}$$

Parameter	Value	Units	Description
I_{max}^{PMCA}	0.575	$\mu A \cdot cm^{-2}$	Maximum sarcolemmal Ca2+ pump current
K_{Ca}^{PMCA}	0.5	μM	Ca2+ half-saturation constant for sarcolemmal Ca2+ pump
K_{ATP1}^{PMCA}	0.012	mM	First ATP half-saturation constant for sarcolemmal Ca2+ pump
K_{ATP2}^{PMCA}	0.23	mM	Second ATP half-saturation constant for sarcolemmal Ca2+ pump
K_{ADP}^{PMCA}	1.0	mM	ADP inhibition constant for sarcolemmal Ca2+ pump

SERCA calcium pump (Jup)

$$J_{up} = \frac{V_f^{up} f_b - V_r^{up} r_b}{(1 + f_b + r_b) f_{ATP}^{SERCA}}$$

$$f_b = \left(\frac{[Ca^{2+}]_i}{K_{fb}}\right)^{N_{fb}}$$

$$r_b = \left(\frac{[Ca^{2+}]_{NSR}}{K_{rb}}\right)^{N_{rb}}$$

$$f_{ATP}^{SERCA} = K_{m,up}^{ATP} / \left([ATP]_i \cdot Hill\left(K_{i1,up}, [ADP]_i, 1\right)\right) + Hill\left(K_{i2,up}, [ADP]_i, \right)^{-1}$$

Parameter	Value	Units	Description
$V_{max,f}^{SERCA}(V_f^{up})$	0.2989	mM/s	SERCA forward rate parameter
$V_{max,b}^{SERCA}(V_r^{up})$	0.3179	mM/s	SERCA reverse rate parameter
K_f^{SERCA}(K$_{fb}$)	0.24	μM	Forward Ca2+ half-saturation constant of SERCA
K_r^{SERCA}(K$_{rb}$)	1.64269	mM	Reverse Ca2+ half-saturation constant of SERCA
N_f^{SERCA}(N$_{fb}$)	1.4		Forward cooperativity constant of SERCA
N_r^{SERCA}(N$_{rb}$)	1.0		Reverse cooperativity constant of SERCA
$K_{ATP}^{SERCA}(K_{m,up}^{ATP})$	0.01	mM	ATP half-saturation constant for SERCA
$K_{ADP1}^{SERCA}(K_{i1,up})$	0.14	mM	ADP first inhibition constant for SERCA
$K_{ADP2}^{SERCA}(K_{i2,up})$	5.1	mM	ADP second inhibition constant for SERCA

Ca2+ transport and buffering parameters

Symbol	Value	Units	Description
τ_{tr}	574.7	s^{-1}	Time constant for transfer from subspace to myoplasm
τ_{xfer}	9090	s^{-1}	Time constant for transfer from NSR to JSR
K_m^{CMDN}	2.38	μM	Ca2+ half saturation constant for calmodulin
K_m^{CSQN}	0.8	mM	Ca2+ half saturation constant for calsequestrin
h_{trpn}^{+}	100000	$s^{-1}\,\mathrm{mM}^{-1}$	Ca2+ on-rate for troponin high-affinity sites
h_{trpn}^{-}	0.33	s^{-1}	Ca2+ off-rate for troponin high-affinity sites
l_{trpn}^{+}	100000	$s^{-1}\,\mathrm{mM}^{-1}$	Ca2+ on-rate for troponin low-affinity sites
l_{trpn}^{-}	40	s^{-1}	Ca2+ off-rate for troponin low-affinity sites
$\Sigma[HTRPN]$	0.14	mM	Total troponin high-affinity sites
$\Sigma[LTRPN]$	0.07	mM	Total troponin low-affinity sites
$\Sigma[CMDN]$	0.05	mM	Total myoplasmic calmodulin concentration
$\Sigma[CQSN]$	15	mM	Total NSR calsequestrin concentration

ODE for cytosolic calcium

$$\beta_i = Hill((K_m^{CMDN} + [Ca^{2+}]_i)^2, K_m^{CMDN} \cdot [CMDN]_{tot}, 1)$$

$$\beta_{ss} = Hill((K_m^{CMDN} + [Ca^{2+}]_{ss})^2, K_m^{CMDN} \cdot [CMDN]_{tot}, 1)$$

$$\beta_{SR} = Hill\left(\left(K_m^{CSQN} + [Ca^{2+}]_{SR}\right)^2, K_m^{CSQN} \cdot [CSQN]_{tot}, 1\right)$$

$$\frac{d[Ca^{2+}]_i}{dt} = \beta_i\left(J_{xfer}\frac{V_{ss}}{V_{myo}} - J_{up} - J_{trpn} - \left(I_{Ca,b} - 2I_{NaCa} + I_{pCa}\right)\frac{A_{cap}}{2V_{myo}F} + (V_{NaCa} - V_{uni})\frac{V_{mito}}{V_{myo}}\right)$$

$$\frac{d[Ca^{2+}]_{SR}}{dt} = \beta_{SR}\left(J_{up}\frac{V_{myo}}{V_{SR}} - J_{rel}\frac{V_{ss}}{V_{SR}}\right)$$

$$J_{trpn} = \frac{d[HTRPNCa]}{dt} + \frac{d[LTRPNCa]}{dt}$$

$$J_{tr} = \frac{[Ca^{2+}]_{NSR} - [\mathrm{Ca}^{2+}]_{\mathrm{JSR}}}{\tau_{tr}}$$

$$J_{xfer} = \frac{[Ca^{2+}]_{SS} - [\mathrm{Ca}^{2+}]_i}{\tau_{xfer}}$$

$$\frac{d[HTRPNCa]}{dt} = k_{htrpn}^{+}\,[Ca^{2+}]_i\,([HTRPN]_{tot} - [HTRPNCa]) - k_{htrpn}^{-}[HTRPNCa]$$

$$\frac{d[LTRPNCa]}{dt} = k_{ltrpn}^{+}\,[Ca^{2+}]_i\,([LTRPN]_{tot} - [LTRPNCa]) - k_{ltrpn}^{-}\left(1 - \frac{2}{3}\,Force_{Norm}\right)[LTRPNCa]$$

IV. Force Generation

$$\frac{d[P_o]}{dt} = -\left(k_{pn}^{trop} + f_{01}\right)[P_o] + k_{np}^{trop}[N_0] + g_{01}(SL)[P_1]$$
$$\frac{d[P_1]}{dt} = -\left(k_{pn}^{trop} + f_{12} + g_{01}(SL)\right)[P_1] + k_{np}^{trop}[N_1] + f_{01}[P_0] + g_{12}(SL)[P_2]$$
$$\frac{d[P_2]}{dt} = -(f_{23} + g_{12}(SL))[P_2] + f_{12}[P_1] + g_{23}(SL)[P_3]$$
$$\frac{d[P_3]}{dt} = -g_{23}(SL)[P_3] + f_{23}[P_2]$$
$$\frac{d[N_1]}{dt} = k_{pn}^{trop}[P_1] + \left(k_{np}^{trop} + g_{01}^{'}(SL)\right)[N_1]$$
$$[N_0] = 1 - ([N_1] + [P_0] + [P_1] + [P_2] + [P_3])$$
$$f_{01} = 3 \times \mathrm{f_{XB}}$$
$$f_{12} = 10 \times f_{\mathrm{XB}}$$
$$f_{23} = 7 \times \mathrm{f_{XB}}$$
$$g_{01} = 1 \times \mathrm{g_{XB}^{min}}$$
$$g_{12} = 2 \times \mathrm{g_{XB}^{min}}$$
$$g_{23} = 3 \times \mathrm{g_{XB}^{min}}$$
$$g_{01}(\mathrm{SL}) = 1 \times \varphi \times \mathrm{g_{XB}^{min}}$$
$$g_{12}(\mathrm{SL}) = 2 \times \varphi \times \mathrm{g_{XB}^{min}}$$
$$g_{23}(\mathrm{SL}) = 3 \times \varphi \times \mathrm{g_{XB}^{min}}$$
$$\varphi = 1 + \frac{2.3 - \mathrm{SL}}{(2.3 - 1.7)^{1.6}}$$
$$k_{np}^{trop} = k_{pn}^{trop}\left[\frac{[LTRPNCa]}{K_{1/2}^{\mathrm{trop}}\ [LTRPN]_{tot}}\right]^{N^{trop}}$$
$$K_{1/2}^{trop} = \left(1 + \frac{K_{\mathrm{Ca}}^{\mathrm{trop}}}{1.7\ 10^{-3} - 0.8\ 10^{-3}\ \frac{(\mathrm{SL} - 1.7)}{0.6}}\right)^{-1}$$
$$N^{trop} = 3.5 \times \mathrm{SL} - 2.0$$
$$K_{Ca}^{trop} = \frac{k_{ltrpn}^{-}}{k_{ltrpn}^{+}}$$
$$\sum PATHS = \mathrm{g_{01}\ g_{12}\ g_{23} + f_{01}\ g_{12}\ g_{23} + f_{01}\ f_{12}\ g_{23} + f_{01}\ f_{12}\ f_{23}}$$
$$P1_{max} = \frac{f_{01}\ \mathrm{g_{12}\ g_{23}}}{\sum \mathrm{PATHS}}$$
$$P2_{max} = \frac{f_{01}\ \mathrm{f_{12}\ g_{23}}}{\sum \mathrm{PATHS}}$$
$$P3_{max} = \frac{f_{01}\ \mathrm{f_{12}\ f_{23}}}{\sum \mathrm{PATHS}}$$
$$Force = \zeta \frac{P_1 + \mathrm{N_1 + 2\ P_2 + 3\ P_3}}{\mathrm{P1_{max} + 2\ P2_{max} + 3\ P3_{max}}}$$
$$Force_{Norm} = \frac{P_1 + \mathrm{N_1 + P_2 + P_3}}{\mathrm{P1_{max} + P2_{max} + P3_{max}}}$$
$$V_{\mathrm{AM}} = \mathrm{V_{AM}^{max}}\left(\frac{f_{01}\ [P_0] + \mathrm{f_{12}}\ [P_1] + \mathrm{f_{23}}\ [P_2]}{f_{01} + \mathrm{f_{12} + f_{23}}}\right)$$
$$\times\left(1 + \frac{K_{\mathrm{M,AM}}^{\mathrm{ATP}}}{[\mathrm{ATP}]_i}\left[1 + \frac{[\mathrm{ADP}]_i}{K_{\mathrm{i,AM}}}\right]\right)^{-1}$$

Parameter	Value	Units	Description
k_{pn}^{trop}	0.04	ms^{-1}	Transition rate from tropomyosin permissive to non-permissive
SL	2.15	µm	Sarcomere length
f_{XB}	0.05	ms^{-1}	Transition rate from weak to strong cross bridge
g_{XB}^{min}	0.1	ms^{-1}	Minimum transition rate from strong to weak cross bridge
ξ	0.1	$N\ mm^{-2}$	Conversion factor normalizing to physiological force
V_{AM}^{max}	7.2×10^{-3}	$mM\ ms^{-1}$	Maximal rate of ATP hydrolysis by myofibrils (AM ATPase)
$K_{M,AM}^{ATP}$	0.03	mM	ATP half saturation constant of AM ATPase
$K_{i,AM}$	0.26	mM	ADP inhibition constant of AM ATPase

V. Cardiac Bioenergetics

Mitochondrial ions

$$\frac{d[Ca^{2+}]_m}{dt} = \delta_{Ca}(J_{uni} - J_{NCLX})$$

$$\frac{d\,\Delta\Psi_m}{\mathrm{dt}} = \frac{V_{\mathrm{He}} + V_{\mathrm{HSDH}} - V_{\mathrm{Hu}} - V_{\mathrm{ANT}} - V_{\mathrm{HLeak}} - V_{\mathrm{NaCa}} - 2\,V_{\mathrm{uni}}}{C_{\mathrm{mito}}}$$

High-energy and inorganic phosphates

$$\frac{d\,[\mathrm{ATP}]_i}{\mathrm{dt}} = J_{\mathrm{ANT}}\frac{V_{\mathrm{mito}}}{V_{\mathrm{myo}}} - V_{\mathrm{CK}}^{\mathrm{mito}} - V_{\mathrm{AM}} - \frac{1}{2}J_{\mathrm{up}} - (I_{\mathrm{pCa}} + I_{\mathrm{NaK}})\frac{A_{cap}}{V_{myo}F}$$

$$\frac{d\,[\mathrm{ATP}]_{\mathrm{ic}}}{\mathrm{dt}} = -V_{\mathrm{CK}}^{\mathrm{cyto}} - V_{\mathrm{ATPase}}^{\mathrm{cyto}}$$

$$\frac{d\,[\mathrm{CrP}]_i}{\mathrm{dt}} = V_{\mathrm{CK}}^{\mathrm{mito}} - V_{\mathrm{tr}}^{\mathrm{CrP}}$$

$$\frac{d\,[\mathrm{CrP}]_{\mathrm{ic}}}{\mathrm{dt}} = V_{\mathrm{tr}}^{\mathrm{CrP}} + V_{\mathrm{CK}}^{\mathrm{cyto}}$$

$$\frac{d\,[\mathrm{ADP}]_m}{\mathrm{dt}} = J_{ANT} - J_{F1Fo} - J_{SL}$$

$$[ATP]_m = C_A - [\mathrm{ADP}]_m$$

Citric acid cycle

$$\frac{d[ISOC]}{dt} = J_{ACO} - J_{IDH}$$
$$\frac{d[\alpha KG]}{dt} = J_{IDH} - J_{KGDH} + J_{AAT}$$
$$\frac{d[SCoA]}{dt} = J_{KGDH} - J_{SL}$$
$$\frac{d[SUC]}{dt} = J_{SL} - J_{SDH}$$
$$\frac{d[FUM]}{dt} = J_{SDH} - J_{FH}$$
$$\frac{d[MAL]}{dt} = J_{FH} - J_{MDH}$$
$$\frac{d[OAA]}{dt} = J_{MDH} - J_{CS} - J_{AAT}$$
$$\frac{d[NADH]_m}{dt} = -V_{O_2} + J_{IDH} + J_{KGDH} + J_{MDH}$$

TCA cycle rates

Citrate synthase (CS)

$$J_{CS} = \frac{k_{cat} E_T AB}{(1+A)(1+B)}$$
$$A = [AcCoA]/K_m^{AcCoA}$$
$$B = [OAA]/K_m^{OAA}$$

Parameter	Value	Unit	Description
k_{cat}	0.23523	s^{-1}	Catalytic constant
E_T	0.4	mM	Enzyme concentration of CS
K_m^{AcCoA}	0.0126	mM	Michaelis constant for AcCoA
K_m^{OAA}	6.4E-4	mM	Michaelis constant for OAA
$[AcCoA]$	1	mM	Acetyl CoA concentration
k_{cat} (cell)	0.15891	s^{-1}	Catalytic constant (cellular model)

Aconitase (ACO)

$$J_{ACO} = k_f\left([CIT] - [ISOC]/K_{eq}\right)$$
$$[CIT] = \Sigma_{CAC} - [ISOC] - [\alpha KG] - [SCoA] - [SUC] - [FUM] - [MAL] - [OAA]$$

Parameter	Value	Unit	Description
k_f	0.11688	s^{-1}	Forward rate constant of ACO
K_{eq}	2.22	-	Equilibrium constant of ACO
Σ_{CAC}	1.300	mM	Sum of TCA cycle intermediates
k_f (cell)	0.078959	s^{-1}	Forward rate constant (cellular model)

Isocitrate dehydrogenase, NADH-producing (IDH)

$$J_{IDH} = \frac{k_{cat}E_T AB}{f_H AB + f_i B + f_a A + f_a f_i}$$
$$f_H = 1 + \frac{[H^+]_m}{K_{H1}} + \frac{K_{H2}}{[H^+]_m}$$
$$A = [NAD]/K_{NAD}$$
$$B = ([ISOC]/K_{ISOC})^n$$
$$f_a = \frac{K_A}{K_A + [ADP]_m}\frac{K_{CA}}{K_{CA} + [Ca^{2+}]_m}$$
$$f_i = 1 + \frac{[NADH]}{K_{NADH}}$$

Parameter	Value	Unit	Description
k_{cat}	11880	s^{-1}	Rate constant of IDH
E_T	0.109	mM	Concentration of IDH
K_{H1}	1E-6	mM	Ionization constant of IDH
K_{H2}	9E-4	mM	Ionization constant of IDH
K_{NAD}	0.923	mM	Michaelis constant for NAD
K_{ISOC}	1.520	mM	Michaelis constant for isocitrate
n	2	-	Cooperativity for isocitrate
K_A	0.62	mM	Activation constant by ADP
K_{CA}	5E-4	mM	Activation constant for calcium
K_{NADH}	0.19	mM	Inhibition constant by NADH
k_{cat} (cell)	535	s^{-1}	Rate constant (cellular model)

Alpha-ketoglutarate dehydrogenase (KGDH)

$$\begin{aligned}
J_{KGDH} &= \frac{k_{cat}E_T AB}{f_H AB + f_a(A+B)} \\
f_H &= 1 + \frac{[H^+]_m}{K_{H1}} + \frac{K_{H2}}{[H^+]_m} \\
A &= [NAD]/K_{NAD} \\
B &= ([\alpha KG]/K_{AKG})^n \\
f_a &= \frac{K_{MG}}{K_{MG} + [Mg^{2+}]_m} \frac{K_{CA}}{K_{CA} + [Ca^{2+}]_m}
\end{aligned}$$

Parameter	Value	Unit	Description
k_{cat}	13.2	s^{-1}	Rate constant of KGDH
E_T	0.5	mM	Concentration of KGDH
K_{H1}	4E-5	mM	Ionization constant of KGDH
K_{H2}	7E-5	mM	Ionization constant of KGDH
K_{NAD}	38.7	mM	Michaelis constant for NAD
K_{AKG}	30	mM	Michaelis constant for αKG
n	1.2	-	Hill coefficient for αKG
K_{MG}	0.0308	mM	Activation constant for Mg
K_{CA}	1.5E-4	mM	Activation constant for Ca
k_{cat} (cell)	17.9	s^{-1}	Rate constant (cellular model)

Succinate-CoA ligase (SL)

$$\begin{aligned}
J_{SL} &= k_f\left([SCoA][ADP]_m[Pi]_m - [SUC][ATP]_m[CoA]/K_{eq}^{app}\right) \\
K_{eq}^{app} &= K_{eq}\frac{P_{SUC}P_{ATP}}{P_{Pi}P_{ADP}}
\end{aligned}$$

Parameter	Value	Unit	Description
k_f	2.8E-5	mM s^{-1}	Forward rate constant of SL
K_{eq}	3.115	-	Equilibrium constant of SL
[CoA]	0.020	mM	Coenzyme A concentration
k_f (cell)	2.84E-5	mM s^{-1}	Forward rate constant (cellular model)

Succinate dehydrogenase (SDH)

$$J_{SDH} = \frac{\mathrm{k}_{\mathrm{cat}}^{\mathrm{SDH}}\ \mathrm{E}_{T}^{\mathrm{SDH}}}{1 + \left(\frac{K_M^{\mathrm{Suc}}}{[\mathrm{Suc}]}\right)\left(1 + \frac{[\mathrm{OAA}]}{K_{\mathrm{i,sdh}}^{\mathrm{OAA}}}\right)\left(1 + \frac{[\mathrm{FUM}]}{K_i^{\mathrm{FUM}}}\right)}$$

Parameter	Value	Unit	Description
k_{cat}^{SDH}	3.0	s^{-1}	Rate constant of SDH
E_T^{SDH}	0.5	mM	SDH enzyme concentration
K_M^{Suc}	0.03	mM	Michaelis constant for succinate

Fumarate hydratase (FH)

$$J_{FH} = k_f\left([FUM] - [MAL]/K_{eq}\right)$$

Parameter	Value	Unit	Description
k_f	8.3	s^{-1}	Forward rate constant
K_{eq}	1.0	-	Equilibrium constant
k_f (cell)	8.4	s^{-1}	Forward rate constant (cellular model)

Malate dehydrogenase (MDH)

$$\begin{aligned}
J_{MDH} &= \frac{k_{cat}E_T AB f_a f_i}{(1 + A)(1 + B)} \\
A &= \frac{[MAL]}{K_{MAL}} \frac{K_{OAA}}{K_{OAA} + [OAA]} \\
B &= [NAD]/K_{NAD} \\
f_a &= k_{offset} + \left(1 + \frac{[H^+]_m}{K_{H1}}\left(1 + \frac{[H^+]_m}{K_{H2}}\right)\right)^{-1} \\
f_i &= \left(1 + \frac{K_{H3}}{[H^+]_m}\left(1 + \frac{K_{H4}}{[H^+]_m}\right)\right)^{2}
\end{aligned}$$

Parameter	Value	Units	Description
k_{cat}	124.2	s^{-1}	Rate constant
E_T	0.154	mM	
K_{H1}	1.131E-5	mM	Ionization constant
K_{H2}	26.7	mM	Ionization constant
K_{H3}	6.68E-9	mM	Ionization constant
K_{H4}	5.62E-6	mM	Ionization constant

Parameter	Value	Units	Description
k_{offset}	0.0399		Offset of MDH pH activation factor
K_{NAD}	0.2244	mM	Michaelis constant for NAD
K_{MAL}	1.493	mM	Michaelis constant for malate
K_{OAA}	0.031	mM	Inhibition constant for oxaloacetate
k_{cat} (cell)	125.9	s^{-1}	Rate constant for cellular model

Aspartate aminotransferase (AAT)

$$J_{AAT} = k_f[OAA][GLU]\frac{k_{ASP}K_{eq}}{k_{ASP}K_{eq} + k_f[\alpha KG]}$$

Parameter	Value	Units	Description
k_f	21.4	s^{-1}	Forward rate constant
k_{ASP}	0.0015	s^{-1}	Rate constant of aspartate consumption
K_{eq}	6.6		Equilibrium constant
[GLU]	30.000	mM	Glutamate concentration
k_f (cell)	21.7	s^{-1}	Forward rate constant (cellular model)

Oxidative phosphorylation reaction rates

$$V_{O_2} = 0.5\,\rho^{\mathrm{res}} \frac{\left(r_a + \mathrm{r_{c1}}\, \mathrm{e}^{\left(\frac{6\mathrm{F}\,\Delta\Psi_B}{\mathrm{R\,T}}\right)}\right) e^{\left(\frac{A_{res}\,\mathrm{F}}{\mathrm{R\,T}}\right)} - r_a\, \mathrm{e}^{\left(\frac{\mathrm{g}\,6\,\mathrm{F}\,\Delta\mu_H}{\mathrm{R\,T}}\right)} + \mathrm{r_{c2}}\, e^{\left(\frac{A_{res}\,\mathrm{F}}{\mathrm{R\,T}}\right)}\, \mathrm{e}^{\left(\frac{\mathrm{g}\,6\,\mathrm{F}\,\Delta\mu_H}{\mathrm{R\,T}}\right)}}{\left(1 + \mathrm{r_1}\, \mathrm{e}^{\left(\frac{\mathrm{F}\,\mathrm{A_{res}}}{\mathrm{R\,T}}\right)}\right) e^{\left(\frac{6\,\mathrm{F}\Delta\Psi_B}{\mathrm{R\,T}}\right)} + \left(r_2 + \mathrm{r_3}\, \mathrm{e}^{\left(\frac{\mathrm{F}\,\mathrm{A_{res}}}{\mathrm{R\,T}}\right)}\right) \mathrm{e}^{\left(\frac{\mathrm{g}\,6\,\mathrm{F}\,\Delta\mu_H}{\mathrm{R\,T}}\right)}}$$

$$V_{He} = 6\,\rho^{\mathrm{res}} \frac{\left(r_a\, e^{\left(\frac{A_{res}\,\mathrm{F}}{\mathrm{R\,T}}\right)} - (r_a + \mathrm{r}_b)\, \mathrm{e}^{\left(\frac{\mathrm{g}\,6\mathrm{F}\,\Delta\mu_H}{\mathrm{R\,T}}\right)}\right)}{\left(1 + \mathrm{r_1}\, \mathrm{e}^{\left(\frac{\mathrm{F}\,\mathrm{A_{res}}}{\mathrm{R\,T}}\right)}\right) e^{\left(\frac{6\,\mathrm{F}\Delta\Psi_B}{\mathrm{R\,T}}\right)} + \left(r_2 + \mathrm{r_3}\, \mathrm{e}^{\left(\frac{\mathrm{F}\,\mathrm{A_{res}}}{\mathrm{R\,T}}\right)}\right) \mathrm{e}^{\left(\frac{\mathrm{g}\,6\,\mathrm{F}\,\Delta\mu_H}{\mathrm{R\,T}}\right)}}$$

$$A_{res} = \frac{\mathrm{R\,T}}{F} \ln\left(K_{\mathrm{res}} \sqrt{\frac{[\mathrm{NADH}]}{[\mathrm{NAD^+}]}}\right)$$

$$[NAD^+] = C_{PN} - [\,\mathrm{NADH}]$$

$$V_{HSDH} = 4\,\rho^{\mathrm{res}(F)} \frac{\left(r_a\, e^{\left(\frac{A_{res(F)}\,\mathrm{F}}{\mathrm{R\,T}}\right)} - (r_a + \mathrm{r}_b)\, \mathrm{e}^{\left(\frac{\mathrm{g}\,4\mathrm{F}\,\Delta\mu_H}{\mathrm{R\,T}}\right)}\right)\frac{1}{1 + \frac{[OAA]}{K_i^{OAA}}}}{\left(1 + \mathrm{r_1}\, \mathrm{e}^{\left(\frac{\mathrm{F}\,\mathrm{A_{res(\mathit{F})}}}{\mathrm{R\,T}}\right)}\right) e^{\left(\frac{4\,\mathrm{F}\Delta\Psi_B}{\mathrm{R\,T}}\right)} + \left(r_2 + \mathrm{r_3}\, \mathrm{e}^{\left(\frac{\mathrm{F}\,\mathrm{A_{res(\mathit{F})}}}{\mathrm{R\,T}}\right)}\right) \mathrm{e}^{\left(\frac{\mathrm{g}\,4\,\mathrm{F}\,\Delta\mu_H}{\mathrm{R\,T}}\right)}}$$

$$A_{res(F)} = \frac{\mathrm{R\,T}}{F} \ln\left(K_{\mathrm{res}(F)} \sqrt{\frac{[\mathrm{SUC}]}{[\mathrm{FUM}]}}\right)$$

$$V_{ATPase} = -\rho^{\mathrm{F1}} \frac{\left(10^2 p_a + \mathrm{p_{c1}}\, \mathrm{e}^{\left(\frac{3\,\mathrm{F}\,\Delta\Psi_B}{\mathrm{R\,T}}\right)}\right) e^{\left(\frac{A_{F1}\,\mathrm{F}}{\mathrm{R\,T}}\right)} - \left(p_a\, \mathrm{e}^{\left(\frac{3\,\mathrm{F}\,\Delta\mu_H}{\mathrm{R\,T}}\right)} + \mathrm{p_{c2}}\, e^{\left(\frac{A_{F1}\,\mathrm{F}}{\mathrm{R\,T}}\right)} \mathrm{e}^{\left(\frac{3\,\mathrm{F}\,\Delta\mu_H}{\mathrm{R\,T}}\right)}\right)}{\left(1 + \mathrm{p_1}\, \mathrm{e}^{\left(\frac{\mathrm{F\,A_{F1}}}{\mathrm{R\,T}}\right)}\right) e^{\left(\frac{3\,\mathrm{F}\Delta\Psi_B}{\mathrm{R\,T}}\right)} + \left(p_2 + \mathrm{p_3}\, \mathrm{e}^{\left(\frac{\mathrm{F\,A_{F1}}}{\mathrm{R\,T}}\right)}\right) \mathrm{e}^{\left(\frac{3\,\mathrm{F}\,\Delta\mu_H}{\mathrm{R\,T}}\right)}}$$

$$V_{Hu} = -3\,\rho^{\mathrm{F1}} \frac{10^2 p_a \left(1 + \mathrm{e}^{\left(\frac{\mathrm{F\,A_{F1}}}{\mathrm{R\,T}}\right)}\right) - (p_a + \mathrm{p}_b)\, \mathrm{e}^{\left(\frac{3\,\mathrm{F}\,\Delta\mu_H}{\mathrm{R\,T}}\right)}}{\left(1 + \mathrm{p_1}\, \mathrm{e}^{\left(\frac{\mathrm{F\,A_{F1}}}{\mathrm{R\,T}}\right)}\right) e^{\left(\frac{3\,\mathrm{F}\Delta\Psi_B}{\mathrm{R\,T}}\right)} + \left(p_2 + \mathrm{p_3}\, \mathrm{e}^{\left(\frac{\mathrm{F\,A_{F1}}}{\mathrm{R\,T}}\right)}\right) \mathrm{e}^{\left(\frac{3\,\mathrm{F}\,\Delta\mu_H}{\mathrm{R\,T}}\right)}}$$

$$A_{F1} = \frac{\mathrm{R\,T}}{F} \ln\left(K_{\mathrm{F1}} \frac{[\mathrm{ATP}]_m}{[ADP]_m\, \mathrm{Pi}}\right)$$

$$V_{Hleak} = \mathrm{g}_H\, \Delta\mu_H$$

$$\Delta\mu_H = -2.303 \frac{\mathrm{R\,T}}{F} \Delta\mathrm{pH} + \Delta\Psi_m$$

Cytosolic metabolic reaction rates

$$V_{ANT} = \mathrm{V_{maxANT}} \frac{0.75\left(1 - \frac{0.25\,[\mathrm{ATP}]_i \times 0.45\,[\mathrm{ADP}]_m}{0.17\,[\mathrm{ADP}]_i \times 0.025\,[\mathrm{ATP}]_m}\right)\left(e^{-\frac{F}{RT}\Delta\Psi_m}\right)}{\left(1 + \frac{0.25\,[\mathrm{ATP}]_i}{0.225\,[\mathrm{ADP}]_i}\, \mathrm{e}\left(\frac{-\mathrm{h^{ANT}}\,\mathrm{F}\,\Delta\Psi_m}{\mathrm{RT}}\right)\right)\left(1 + \frac{0.45\,[\mathrm{ADP}]_m}{0.025\,[\mathrm{ATP}]_m}\right)}$$

$$V_{CK}^{cyto} = k_{CK}^{cyto}\left([ATP]_{ic}\,[Cr]_{ic} - \frac{[ADP]_{ic}\,[CrP]_{ic}}{K_{EQ}}\right)$$

$$V_{CK}^{mito} = k_{CK}^{mito}\left([ATP]_i\,[Cr]_i - \frac{[ADP]_i\,[CrP]_i}{K_{EQ}}\right)$$

$$V_{\mathrm{tr}}^{\mathrm{CrP}} = \mathrm{k_{tr}^{Cr}}([\mathrm{CrP}]_i - [\mathrm{CrP}]_{\mathrm{ic}})$$

Parameter	Value	Units	Description
r_a	6.394×10^{-13}	ms^{-1}	Sum of products of rate constants
r_b	1.762×10^{-16}	ms^{-1}	Sum of products of rate constants
r_{c1}	2.656×10^{-22}	ms^{-1}	Sum of products of rate constants
r_{c2}	8.632×10^{-30}	ms^{-1}	Sum of products of rate constants
r_1	2.077×10^{-18}		Sum of products of rate constants
r_2	1.728×10^{-9}		Sum of products of rate constants
r_3	1.059×10^{-26}		Sum of products of rate constants
ρ^{res}	3.0×10^{-3}	mM	Concentration of electron carriers (respiratory complexes I-III-IV)

Parameter	Value	Units	Description
K_{res}	1.35×10^{18}		Equilibrium constant of respiration
$\rho^{res(F)}$	3.75×10^{-4}	mM	Concentration of electron carriers (respiratory complexes II-III-IV)
$\Delta\Psi_B$	50	mV	Phase boundary potential
g	0.85		Correction factor for voltage
$K_{res(F)}$	5.765×10^{13}		Equilibrium constant of $FADH_2$ oxidation
K_i^{OAA}	0.15		Inhibition constant for OAA
p_a	1.656×10^{-8}	ms^{-1}	Sum of products of rate constants
p_b	3.373×10^{-10}	ms^{-1}	Sum of products of rate constants
p_{c1}	9.651×10^{-17}	ms^{-1}	Sum of products of rate constants
p_{c2}	4.585×10^{-17}	ms^{-1}	Sum of products of rate constants
p_1	1.346×10^{-8}		Sum of products of rate constants
p_2	7.739×10^{-7}		Sum of products of rate constants
p_3	6.65×10^{-15}		Sum of products of rate constants
ρ^{F1}	1.5	mM	Concentration of F_1F_0-ATPase
K_{F1}	1.71×10^{6}		Equilibrium constant of ATP hydrolysis
Pi	2.0	mM	Inorganic phosphate concentration
C_A	1.5	mM	Total sum of mitochondrial adenine nucleotides
V_{maxANT}	0.025	mM ms^{-1}	Maximal rate of the ANT
h^{ANT}	0.5		Fraction of $\Delta\Psi_m$
g_H	1.0×10^{-8}	mM ms^{-1} mV^{-1}	Ionic conductance of the inner membrane
ΔpH	-0.6	pH units	pH gradient across the mitochondrial inner membrane
C_{PN}	10.0	mM	Total sum of mitochondrial pyridine nucleotides
δ_{Ca}	0.0003		Mitochondrial free calcium fraction
k_{CK}^{cyto}	1.4×10^{-4}	ms^{-1}	Forward rate constant of cytoplasmic CK
k_{CK}^{mito}	1.33×10^{-6}	ms^{-1}	Forward rate constant of mitochondrial CK

Parameter	Value	Units	Description
k_{tr}^{Cr}	2.0×10^{-3}	ms^{-1}	Transfer rate constant of CrP
K_{EQ}	0.0095		Equilibrium constant of CK
V_{ATPase}^{cyto}	$1.0\ 10^{-5}$	$mM\ ms^{-1}$	Constitutive cytosolic ATP consumption rate

Mitochondrial Ca^{2+} handling rates

$$V_{uni} = V_{\max}^{\text{uni}} \frac{\frac{[Ca^{2+}]_i}{K_{trans}}\left(1 + \frac{[Ca^{2+}]_i}{K_{trans}}\right)^3 \frac{2\,\mathrm{F}\,(\Delta\Psi_m - \Delta\Psi^o)}{R\,\mathrm{T}}}{\left(\left(1 + \frac{[Ca^{2+}]_i}{K_{trans}}\right)^4 + \frac{L}{\left(1 + \frac{[Ca^{2+}]_i}{K_{act}}\right)^{n_a}}\right)\left(1 - \mathrm{e}^{\left\{\frac{-2\,\mathrm{F}\,(\Delta\Psi_m - \Delta\Psi^o)}{R\,\mathrm{T}}\right\}}\right)}$$

$$V_{NaCa} = V_{\max}^{\text{NaCa}} \frac{e^{\left(\frac{b\,F\,(\Delta\Psi_m - \Delta\Psi^\circ)}{R\,T}\right)} e^{\left(ln \frac{[Ca^{2+}]_m}{[Ca^{2+}]_i}\right)}}{\left(1 + \frac{K_{Na}}{[Na^+]_i}\right)^n \left(1 + \frac{K_{Ca}}{[Ca^{2+}]_m}\right)}$$

Parameter	Value	Unit	Description
V_{max}^{uni}	0.0275	$mM\ ms^{-1}$	Vmax uniport Ca^{2+} transport
$\Delta\Psi^\circ$	91	mV	Offset membrane potential
K_{act}	3.8×10^{-4}	mM	Activation constant
K_{trans}	0.019	mM	K_d for translocated Ca^{2+}
L	110.0		Keq for conformational transitions in uniporter
n_a	2.8		Uniporter activation cooperativity
V_{max}^{NaCa}	0.8×10^{-4}	$mM\ ms^{-1}$	Vmax of Na^+/Ca^{2+} antiporter
b	0.5		$\Delta\Psi_m$ dependence of Na^+/Ca^{2+} antiporter
K_{Na}	9.4	mM	Antiporter Na^+ constant
K_{Ca}	3.75×10^{-4}	mM	Antiporter Ca^{2+} constant
n	3		Na^+/Ca^{2+} antiporter cooperativity
δ	3.0×10^{-4}		Fraction of free $[Ca^{2+}]_m$

State variables initial conditions

Symbol	Description	HF normal ATP	HF low ATP
[ATP]i	EC coupling linked ATP concentration	6.90E+00	3.70E+00
V	Sarcolemmal membrane potential	-8.57E+01	-8.54E+01
P_{C1}	Fraction of RyR channels in P_{C1} state	2.49E-01	2.36E-01
P_{C2}	Fraction of RyR channels in P_{C2} state	7.50E-01	7.63E-01
P_{O2}	Fraction of RyR channels in P_{O2} state	9.42E-09	9.88E-09
m_{Na}	Sodium channel activation gate	3.28E-02	3.27E-02
n_{Na}	Sodium channel inactivation gate	9.87E-01	9.86E-01
j_{Na}	Sodium channel slow inactivation gate	9.92E-01	9.91E-01
xKs	Potassium channel activation gate	3.68E-02	4.36E-02
O	L-type Ca^{2+} channel open – mode normal	8.10E-12	2.45E-14
O_{Ca}	L-type Ca^{2+} channel open – mode Ca	0.00E+00	0.00E+00
y	ICa inactivation gate	4.89E-01	4.82E-01
$[K^+]_i$	Intracellular K^+ concentration	1.48E+02	1.47E+02
$[Na^+]_i$	Intracellular Na^+ concentration	7.35E+00	7.58E+00
$[Ca^{2+}]_i$	Intracellular Ca^{2+} concentration	9.80E-05	1.03E-04
$[ADP]_m$	Mitochondrial ADP concentration	3.87E-01	5.21E-01
$\Delta\Psi_m$	Inner mitochondrial membrane potential	1.56E+02	1.55E+02
[NADH]	Mitochondrial NADH concentration	7.01E+00	7.18E+00
[OAA]	Oxalacetate concentration (mitochondrial)	3.67E-07	3.89E-07
[αKG]	α-ketoglutarate concentration (mitochondrial)	1.41E-03	1.45E-03
[SCoA]	Succinyl CoA concentration (mitochondrial)	5.88E-02	4.63E-02
[Suc]	Succinate concentration (mitochondrial)	1.50E-03	1.60E-03
[FUM]	Fumarate concentration (mitochondrial)	6.51E-02	6.92E-02
[MAL]	Malate concentration (mitochondrial)	3.09E-02	3.30E-02
[ISOC]	Isocitrate concentration (mitochondrial)	5.74E-01	5.78E-01

$[Ca^{2+}]_m$	Mitochondrial free Ca^{2+} concentration	3.45E-04	3.91E-04
$[Ca^{2+}]_{NSR}$	Network SR Ca^{2+} concentration	4.06E-01	3.80E-01
$[Ca^{2+}]_{JSR}$	Junctional SR Ca^{2+} concentration	4.06E-01	3.78E-01
$[Ca^{2+}]_{SS}$	Ca^{2+} concentration in the subspace	1.71E-04	1.73E-04
$[N_1]$	Nonpermissive tropomyosyn with 1 cross bridges	1.05E-03	1.78E-03
$[P_0]$	Permissive tropomyosyn with 0 cross bridges	7.71E-04	1.23E-03
$[P_1]$	Permissive tropomyosyn with 1 cross bridges	8.40E-04	1.38E-03
$[P_2]$	Permissive tropomyosyn with 2 cross bridges	1.66E-03	2.75E-03
$[P_3]$	Permissive tropomyosyn with 3 cross bridges	1.47E-03	2.45E-03
[LTRPNCa]	Ca^{2+} bound to low affinity troponin sites	1.39E-02	1.46E-02
[HTRPNCa]	Ca^{2+} bound to high affinity troponin sites	1.36E-01	1.36E-01
C_1	L-type Ca^{2+} channel closed – mode normal	1.16E-05	1.20E-05
C_{Ca0}	L-type Ca^{2+} channel closed – mode Ca	2.74E-02	2.97E-02
C_0	L-type Ca^{2+} channel closed – mode normal	9.73E-01	9.70E-01
C_2	L-type Ca^{2+} channel closed – mode normal	5.22E-11	5.59E-11
C_{Ca1}	L-type Ca^{2+} channel closed – mode Ca	1.31E-06	1.47E-06
C_3	L-type Ca^{2+} channel closed – mode normal	1.79E-13	6.67E-16
C_{Ca2}	L-type Ca^{2+} channel closed – mode Ca	2.35E-11	2.73E-11
C_4	L-type Ca^{2+} channel closed – mode normal	1.30E-13	4.05E-16
C_{Ca3}	L-type Ca^{2+} channel closed – mode Ca	-3.20E-18	2.26E-16
C_{Ca4}	L-type Ca^{2+} channel closed – mode Ca	-8.18E-17	4.91E-21
$[CrP]_i$	Mitochondrial linked creatine phosphate concentration	1.04E+01	5.64E+00
$[CrP]_{ic}$	Cytosolic creatine phosphate concentration	1.12E+01	7.30E+00
$[ATP]_{ic}$	Cytosolic ATP concentration not linked to EC coupling	6.90E+00	3.70E+00
ASP	Asparatate concentration (mitochondrial)	4.88E-02	4.88E-02
$[N_0]$	Nonpermissive tropomyosyn with 0 cross bridges	9.94E-01	9.90E-01
